# Association of exhaled carbon monoxide with risk of cardio-cerebral-vascular disease in the China Kadoorie Biobank cohort study

**DOI:** 10.1038/s41598-020-76353-2

**Published:** 2020-11-11

**Authors:** Gaokun Qiu, Kuai Yu, Canqing Yu, Wending Li, Jun Lv, Yu Guo, Zheng Bian, Ling Yang, Yiping Chen, Zhengming Chen, Frank B. Hu, Liming Li, Tangchun Wu

**Affiliations:** 1 Department of Occupational and Environmental Health, Key Laboratory of Environment and Health, Ministry of Education and State Key Laboratory of Environmental Health (Incubating), School of Public Health, Tongji Medical College, Huazhong University of Science and Technology, 13 Hongkong Rd, Wuhan, 430030 Hubei People’s Republic of China; 2 Department of Epidemiology and Biostatistics, School of Public Health, Peking University Health Science Center, 38 Xueyuan Road, Beijing, 100191 People’s Republic of China; 3 Chinese Academy of Medical Sciences, Beijing, People’s Republic of China; 4 Clinical Trial Service Unit & Epidemiological Studies Unit (CTSU), Nuffield Department of Population Health, University of Oxford, Oxford, UK; 5 Department of Nutrition and Department of Epidemiology, Harvard T.H. Chan School of Public Health, Boston, MA 02115 USA

**Keywords:** Cardiology, Diseases

## Abstract

Exhaled carbon monoxide (COex) level has been proposed as a noninvasive and easily-obtainable cardiovascular risk marker, however, with limited prospective evidence, and its association with stroke risk has been rarely explored. Measurements of COex were performed during 2004–2008 baseline examinations in the China Kadoorie Biobank study among 512,891 adults aged 30–79 years from 10 diverse study areas. After excluding participants with baseline cardiopulmonary diseases, stroke and cancer, 178,485 men and 267,202 women remained. Cox regression yielded hazard ratios (HRs) and 95% confidence intervals (CIs) for risk of cardio-cerebral-vascular disease (CCVD) associated with COex levels, with sequential addition of adjustment for proxy variables for CO exposure, including study area indexing ambient CO variations at large, and smoking and solid fuel use, apart from adjusting for traditional cardiovascular risk factors. During 7-year follow-up, we documented 1744 and 1430 major coronary events (myocardial infarction plus fatal ischemic heart disease), 8849 and 10,922 ischemic strokes, and 2492 and 2363 hemorrhagic strokes among men and women, respectively. The HRs with 95% CIs comparing the highest with lowest COex quintile were 2.15 [1.72, 2.69] for major coronary events, 1.65 [1.50, 1.80] for ischemic stroke, and 1.35 [1.13, 1.61] for hemorrhagic stroke among men, while among women higher associated risk was only observed for major coronary events (1.64 [1.35, 2.00]) and ischemic stroke (1.87 [1.73, 2.01]). The elevated risks were consistent when COex level was over 3 ppm. However, these associations were all attenuated until null by sequential addition of stratification by study areas, and adjustments of smoking and solid fuel use. Nevertheless, the association with ischemic stroke was maintained among the subgroup of male smokers even with adjustment for the depth and amount of cigarette smoking (HR [95% CI]: 1.37 [1.06, 1.77]), while a negative association with hemorrhagic stroke also appeared within this subgroup. Higher COex level (over 3 ppm) was associated with elevated risk of ischemic CCVD, but not independently of CO exposure. Our finding suggests that, though not an independent risk factor, COex could potentially provide a cost-effective biomarker for ischemic cardio-cerebral-vascular risk, given that CO exposure is ubiquitous.

## Introduction

Carbon moxide (CO), an air pollutant ubiquitously and abundantly distributed in ambient air^1^ and residential microenvironments^2^, is stemming from incomplete combustion of carbonaceous materials such as fossil fuels and biomass^2^, and also exists in large quantities in cigarette smoke^3^. In human body, CO originates mainly from exogenous exposure to ambient and household air pollution, as well as cigarette smoking^4^, and also produced endogenously, though in much smaller amount, through the enzymatic degradation of heme^5,6^. Despite mixed origination, CO is eliminated almost exclusively through the lungs^7^, and CO levels in exhaled breath (COex) could be closely reflecting integrated levels of CO exposure from various sources.There is evidence suporting the association between COex level and CO exposure from household air pollution^8,9^, and cigarette consumption^8,10^, with the latter being the primary source of short-term exposure^4^, though it has not been explored for COex level as indexing CO exposure from ambient air pollution.

Several prospective studies have reported associations between COex levels and risk of cardio-cerebral-vascular disease (CCVD)^11–16^, and it has been repeatedly proposed that COex level might provide an informative, noninvasive and cost-effective biomarker for cardiometabolic risk^7,17^. However, inconsistencies and uncertainty remain within these studies. The Framingham study reported positive associations of COex levels with the risk of developing cardiovascular disease (CVD)^11^ and stroke/transient ischemic attack^12^, and with the risk of progressing from subclinical to clinical CVD^13^, independently of smoking status. The Scottish Heart Health Extended Cohort study also found that higher COex level was associated with increased risk of coronary heart disease (CHD), peripheral artery disease (PAD)^14^ and atrial fibrillation (AF)^15^, whereas the adjustment for cigarette equivalent dose moderately attenuated the associations with CHD and PAD while abolished the association with AF. In another study, Berard et al*.* also found that the positive association between COex levels and CVD mortality was abolished by adjustment for smoking status and pack-years^16^. According to these studies, the adjustment of smoking tended to have an appreciable impact, though to an extent not yet determined, on the association between COex level and CCVD risk, which raised the concern about the independency of this association. To address such concern, it entailed adequate adjustment for various sources of CO exposure, including ambient and household air pollution that were accounted for in none of these studies. More importantly, stroke was particularly under-represented among the CCVD endpoints investigated for association with COex level, only included as a component in CVD or combined with transient ischemic attack as a composite outcome^11–13,16^, without being examined individually, and the association with the ischemic and hemorrhagic subtypes of stroke, which had different risk factor profiles^18^, has not been explored.

In the present study, we prospectively investigated the association between COex levels and risk of ischemic stroke, hemorrhagic stroke and major coronary events (including fatal ischemic heart disease and nonfatal myocardial infarction) as individual endpoints in the China Kadoorie Biobank study of 0.5 million adults, with and without adjustment for a number of proxy variables accounting for various sources of CO exposure.

## Materials and methods

### Study population and baseline survey

Full details of the study design, methods and participants of the China Kadoorie Biobank study have been described previously^19,20^. In brief, between June 2004 and July 2008, 512,891 participants aged 30 to 79 years were recruited from 10 study areas across China (five rural and five urban areas). Each participant was administered a laptop-based questionnaire by a trained interviewer to collect information on sociodemographic characteristics, smoking and alcohol consumption, physical activities, household solid fuel use and medical history. Daily level of physical activity was calculated by multiplying the metabolic equivalent tasks (METs) value for a particular type of physical activity by hours spent on that activity per day and summing the MET hours for all activities. Anthropometric measurements such as height, weight, and blood pressure were performed by trained staff. Random blood glucose was measured on-site using the SureStep Plus System (Johnson & Johnson).

In the present study, we excluded 67,204 participants with self-reported physician-diagnosed baseline CHD, stroke, cancer, asthma, chronic obstructive pulmonary disease, emphysema, bronchitis, or tuberculosis, and finally 445,687 participants remained in the analyses. This study complied with the Declaration of Helsinki, and was approved by the ethical review committee of the Chinese Center for Disease Control and Prevention (Beijing, China) and the Oxford Tropical Research Ethics Committee, University of Oxford (UK). All participants had completed a written informed consent form.

### Measurement of exhaled carbon monoxide

COex level was measured twice by a hand-held battery-operated meter (MicroCO meter, CareFusion, UK) and then averaged, as described previously^8^. During measurement, each participant was asked to inhale fully, hold their breath for 20 s, and then blow slowly and fully into the mouthpiece adapter. Expired alveolar gas was entrapped between the mouthpiece valve and sensor, and sensor would detect peak expired concentrations of CO in alveolar gas. Calibration was conducted regularly using a calibration gas of up to 20 ppm CO in air.

### Definition of covariates concerning smoking and solid fuel use

Participants were categorized as current regular smokers if they were smoking daily or almost daily at the survey time, as ex-smokers if they had abstained from regular smoking for at least 6 months, as occasional smokers if they only smoked occasionally, or as never smokers if they had never smoked. Ever smokers were further asked about the types and amount of tobacco they usually smoked, the smoking frequency, and whether having smoked at least 100 cigarettes or equivalents during their lifetime. The number of cigarette equivalents smoked per day was calculated for current and ex-regular smokers including all types of tobacco, with one pipe or one hand-rolled cigarette being treated as equal to 5/3 cigarettes, and one cigar being treated as equal to 2 cigarettes. Regular smokers were also asked about the depth of inhalation, while ex-smokers were asked about the time in years since cessation of smoking. Passive smoking was defined if the participant had been living with a smoker in the same house for at least 6 months at baseline. We also assessed household solid fuel use according to reported fuel use at each participant’s baseline residence. Specifically, solid fuel use for cooking was defined as using coal or wood for cooking, and solid fuel use for heating was defined if the participant used coal or wood for heating and his/her baseline examination took place in winter season. Slow burning of solid fuel was defined if the participant always kept a coal-burning stove under slow burning throughout the day.

### Ascertainment of incident CCVD

All participants were tracked annually through the Disease Surveillance Points system of the Chinese Center for Disease Control and Prevention for vital status against local residential records and health insurance records, with confirmation from residential committees or village administrators. Information on major diseases and hospitalization data were collected through electronic linkages to disease registries, which were obtained through the Chinese National Health Insurance scheme, and the national health insurance claims databases. Cardio-cerebral-vascular events were monitored till December 31, 2013. Fatal and nonfatal events were documented according to the International Classification of Diseases, 10th Revision (ICD-10). The main outcomes examined were major coronary events (myocardial infarction [code I21] and fatal ischemic heart disease [codes I20 to I25]), ischemic stroke (code I63) and hemorrhagic stroke (code I61). For outcome cases with medical records retrieved, the diagnosis adjudication was performed by qualified cardiovascular specialists blinded to study assay, which revealed high accuracy (> 80%) of electronic linkages in identifying CVD cases^20^.

### Statistical analysis

Participants were divided according to COex quintiles (< 2.0, 2.0–3.0, 3.0–5.0, 5.0–11.5 and ≥ 11.5 ppm). We performed Cox regression to estimate hazard ratios (HRs) and 95% confidence intervals (CIs) for risks of developing CCVD in relation to COex levels, and all analysis were performed separately among men and women. Cox models were stratified by age-at-risk (in 5-year intervals), urban residency, and adjusting for traditional cardiovascular risk factors (including BMI, systolic blood pressure, baseline status of diabetes, baseline status of hypertension, alcohol drinking and physical activity, but not including smoking), indicators of socioeconomic status (including education and household income), and the survey season in model 1, stratified by study areas instead of urban residency in model 2, further adjusted for smoking status, passive smoking in model 3, plus household solid fuel use in model 4. We compared adjusted HRs for the first 4 years and for the subsequent years of follow-up, and no evidence of departure from the proportional hazards assumption was observed for all models. Among the adjusted covariates, stratification by study areas was expected to account for regional variations of ambient air pollution at large, which was the primary determinant of long-term CO exposure^4^, while smoking and household solid fuel use were sources of acute exposure to air pollution and major determinants of short-term CO exposure^4^. We then performed subgroup analysis by smoking status with full adjustment as in model 4, except for that smoking was indexed by cigarette equivalents/day and self-reported depth of inhalation among current regular smokers, whether having smoked at least 100 cigarettes or equivalent among occasional smokers, and cigarette equivalents per day, the time in years since smoking cessation and self-reported depth of inhalation among ex-smokers. We also constructed restricted cubic spline (RCS) plots to visually examine the relationship between COex levels and risk of CCVD. We then performed sensitivity analyses by: (1) excluding events occurring within 2 years after baseline survey to scrutinize reverse causality; (2) separate analysis within participants from each study area of the association between COex level and CCVD risk, and meta-analysis of such association; (3) examining the association of COex level with total CCVD death, fatal ischemic events (i.e. deaths from major coronary events or ischemic stroke), and fatal hemorrhagic stroke. Further subgroup analyses were conducted separately among never smokers and current regular smokers according to urban residency and baseline characteristics of traditional cardiovascular risk factors, including age, BMI, physical activity, prevalent hypertension and diabetes, and multiplicative interaction was tested. All analyses were performed using SAS 9.4 (SAS Institute, Cary, NC, USA), and graphs were plotted using R version 3.6.0 (R Foundation), or the SAS software.

## Results

### Baseline characteristics

Table 1 presents the baseline characteristics of the study participants according to COex quintiles. It was consistently observed among men and women that participants with higher COex concentrations were younger, more likely to be rural residents, to smoke and drink regularly, to have higher levels of waist circumference, and participants in the middle COex quintile had the highest glucose levels, and diabetes prevalence (*P*_trend_ < 0.001). The association between COex level and other risk factors showed less clear trend.

**Table 1:** Baseline characteristics of the study population by quintiles of COex levels.

Characteristics	COex quintiles (ppm)											
	Men						Women					
	< 2.0	2.0–3.0	3.0–5.0	5.0–11.5	≥ 11.5	*P*_trend_	< 2.0	2.0–3.0	3.0–5.0	5.0–11.5	≥ 11.5	*P*_trend_
N	11,563	16,358	29,538	50,541	70,485	–	64,625	64,899	66,588	50,139	20,951	–
Age (years)	54.61 (11.13)	53.57 (11.00)	52.68 (11.01)	52.37 (10.69)	49.51 (9.56)	< 0.001	51.24 (10.51)	50.29 (10.11)	50.37 (10.06)	50.45 (10.12)	50.62 (9.96)	< 0.001
Urban residency (%)	44.5%	52.1%	50.7%	40.6%	39.7%	< 0.001	40.7%	48.6%	51.1%	40.7%	19.2%	< 0.001
BMI (kg/m^2^)	23.39 (3.05)	23.75 (3.10)	24.03 (3.18)	23.61 (3.28)	23.17 (3.17)	< 0.001	23.35 (3.29)	23.73 (3.35)	24.00 (3.45)	24.00 (3.44)	24.27 (3.54)	< 0.001
Waist circumference (cm)	80.94 (9.30)	82.17 (9.38)	83.30 (9.59)	82.34 (9.84)	81.70 (9.54)	< 0.001	77.58 (9.21)	786.06 (93.07)	79.40 (9.48)	79.70 (9.37)	80.12 (9.50)	< 0.001
Physical activity (MET-hr/day)	22.23 (14.93)	22.12 (14.87)	22.12 (14.88)	23.22 (15.25)	23.66 (15.51)	< 0.001	23.24 (13.53)	22.25 (13.12)	20.28 (12.31)	18.89 (11.89)	17.28 (11.74)	< 0.001
Current drinkers (%)	69.6%	72.6%	76.4%	77.2%	81.3%	< 0.001	22%	30%	39.1%	49.1%	61.6%	< 0.001
**Smoking status (%)**												
Never smoker	33.1%	29.9%	28.4%	13.1%	2.6%	< 0.001	97.3%	97.2%	96.5%	93.6%	87.4%	< 0.001
Ex-smokers	20.9%	22.2%	19.9%	12.8%	3.2%		1.5%	1.5%	1.8%	2.1%	1.8%	
Occasional smokers	13.9%	20.2%	31.1%	27.2%	7.6%		0.6%	0.6%	0.7%	0.6%	0.3%	
Current regular smokers	21.4%	22.6%	30.1%	63.1%	92.0%		0.6%	0.7%	1.0%	3.6%	10.5%	
**House-hold income (%)**												
≤ 4,999 yuan/ year	9.2%	8.5%	7.8%	8.7%	8.0%	< 0.001	9.9%	9.0%	8.2%	9.7%	13.5%	< 0.001
5,000–9,999 yuan/ year	13.3%	13.4%	14.7%	16.5%	19.0%		20.1%	19.0%	18.3%	19.8%	24.1%	
10,000–19,999 yuan/ year	22.7%	25.3%	26.5%	27.1%	31.4%		24.1%	26.7%	30.7%	34.9%	39.7%	
20,000–34,999 yuan/ year	26.1%	26.9%	27.8%	26.4%	24.6%		25.1%	25.9%	26.7%	23.0%	16.1%	
≥ 35,000 yuan/ year	28.7%	25.9%	23.2%	21.3%	17.1%		20.7%	19.4%	16.1%	12.7%	6.6%	
**Education (%)**												
No formal school	12.7%	8.9%	7.8%	10.2%	6.0%	< 0.001	35.2%	27.5%	19.9%	16.6%	17.4%	< 0.001
Primary school	37.2%	34.4%	31.4%	33.4%	29.8%		31.4%	30.3%	30.0%	31.6%	36.9%	
Middle school	26.2%	30.5%	30.9%	31.7%	38.6%		20.8%	25.2%	28.0%	29.4%	30.1%	
High school	14.7%	16.5%	18.6%	17.0%	19.9%		9.6%	12.7%	16.2%	17.1%	13.1%	
College or university	9.1%	9.7%	11.4%	7.7%	5.7%		3.0%	4.3%	5.9%	5.3%	2.5%	
Systolic blood pressure (mmHg)	134.69 (20.63)	133.63 (19.59)	132.96 (19.50)	132.76 (19.99)	130.63 (19.17)	< 0.001	130.1 (22.01)	128.91 (21.46)	127.84 (21.36)	128.83 (21.74)	132.69 (22.42)	< 0.001
Diastolic blood pressure (mmHg)	79.99 (11.10)	79.85 (11.00)	79.76 (11.14)	79.48 (11.42)	78.44 (11.37)	< 0.001	76.91 (10.76)	76.77 (10.71)	76.34 (10.78)	76.38 (11.04)	77.44 (11.22)	< 0.001
Antihypertensive medication (%)	8.9%	12.2%	19.9%	29.5%	29.4%	< 0.001	23.3%	23.9%	24.6%	19.2%	9.0%	< 0.001
Hypertension (%)	13.5%	12.5%	11.3%	9.5%	6.6%	< 0.001	10.1%	10.0%	10.3%	10.1%	8.8%	< 0.001
Blood glucose (mmol/L)	5.85 (2.05)	5.99 (2.36)	6.08 (2.40)	5.95 (2.32)	5.85 (2.22)	< 0.001	5.97 (2.044)	6.07 (2.18)	6.18 (2.35)	6.17 (2.45)	6.06 (2.49)	< 0.001
Diabetes (%)	5.6%	5.8%	6.1%	5.3%	4.2%	< 0.001	4.8%	5.3%	6.0%	6.0%	5.7%	< 0.001

Data are means (SD) for continuous variables or percentages for categorical variables.

COex, exhaled carbon monoxide.

BMI, body mass index; calculated as weight (kg) / height (m)^2^.

Physical activity was assessed with metabolic equivalent of task value for a day’s work and leisure activities, calculated by multiplying the metabolic equivalent tasks (METs) value for a particular type of physical activity by hours spent on that activity per day and summing the MET hours for all activities.

At the exchange rate as of August 2020, 100 yuan is approximately equal to 14.5 U.S. dollars.

### The association between COex level and CO exposure

At baseline, COex level was dose-dependently associated with cigarette equivalents/day and the depth of inhalation (*P*_trend_ < 0.001), and also higher among participants with household solid fuel use (*P* < 0.001; Supplementary Fig. S1). The pattern of CO exposure differed between genders, and rural/urban residents (Supplementary Fig. S2). Males were more likely to be exposed to cigarette smoking, while females were more likely to be exposed to solid fuel use (*P* < 0.001), which was reported almost exclusively among rural residents (*P* < 0.001).

Baseline levels of COex across the 10 study areas were presented in Supplementary Fig. S3. COex level at baseline was highest in Henan (median [25th to 75th]: 8.5 [5.0–16.0] among never smokers; 20.5 [13.0–29.5] among current regular smokers), and lowest in Haikou among never smokers (1.5 [1.0–2.5]). Ambient CO levels were not available at baseline, and we were not able to examine the correlation between ambient CO and COex levels. As a proof of concept, we examined the correlations of regional median COex levels among never smokers with average ambient CO and PM_2.5_ levels from January, 2014 to December, 2017 in the five urban areas, which was obtained from the National Urban Air Quality Real-time Publishing Platform (https://106.37.208.233:20035/). The four-year average ambient CO levels in the cities of Harbin, Qingdao, Suzhou, Liuzhou and Haikou were 1.03, 0.84, 0.91, 1.06 and 0.66 mg/m^3^, while the corresponding PM_2.5_ levels were 62.22, 47.31, 53.12, 51.05 and 21.34 μg/m^3^, and moderate correlations of regional COex levels with ambient CO and PM_2.5_ were observed (Spearman rank correlation coefficients = 0.500 and 0.600, respectively).

### The association between COex level and future risk of CCVD

During 7.05 ± 1.47 years of follow-up, we documented 1744 and 1430 major coronary events (myocardial infarction plus fatal ischemic heart disease), 8849 and 10,922 ischemic strokes, 2492 and 2363 hemorrhagic strokes among men and women, respectively. Participants in the highest COex quintile had higher risk of major coronary events (HR [95% CI] for the highest vs lowest quintile: 2.15 [1.72, 2.69] among men, and 1.64 [1.35, 2.00] among women) and ischemic stroke (1.65 [1.50, 1.80] among men, and 1.87 [1.73, 2.01] among women) compared with those in the lowest quintile, when stratified by urban residency and adjusting for cardiovascular risk factors not including smoking (Table 2). The associated risks were consistent when COex level was over 3 ppm. After stratification by study areas, the proxy variable for CO exposure from regional ambient air pollution, such associated risks were attenuated but persisted among men (HR [95% CI]: 1.38 [1.09, 1.75] for major coronary events, and 1.23 [1.11, 1.35] for ischemic stroke), and partially maintained among women (1.10 [1.01, 1.20] for ischemic stroke), although further adjustment for other sources of CO exposure (smoking and solid fuel use) still abolished these associations. As for hemorrhagic stroke, increasing risk with higher COex level was only observed among men (HR [95% CI]: 1.35 [1.13, 1.61]), and no longer existed upon additional adjustments for CO exposure variables. In the analysis across subgroups of smoking status (Table 3 and Supplementary Table S1), the association with ischemic stroke was persistent among the subgroup of male smokers after adjustment for variables of CO exposure (HR [95% CI]: 1.37 [1.06, 1.77]), while a negative association with hemorrhagic stroke also appeared within this subgroup (0.68 [0.49, 0.96]).

**Table 2:** The association between COex levels and future risk of CCVD.

Outcomes	COex quintiles (ppm)									
	Men					Women				
	< 2.0	2.0–3.0	3.0–5.0	5.0–11.5	≥ 11.5	< 2.0	2.0–3.0	3.0–5.0	5.0–11.5	≥ 11.5
**Major coronary events**										
No. of events (%)	91 (0.1%)	145 (0.1%)	271 (0.2%)	535 (0.3%)	702 (0.4%)	303 (0.1%)	268 (0.1%)	340 (0.1%)	339 (0.1%)	180 (0.1%)
Incidence rate	51.4 (41.5, 63.6)	62.0 (52.1, 73.7)	68.5 (60.2, 78.0)	84.7 (76.8, 93.4)	110.4 (101.4, 120.2)	26.7 (23.3, 30.7)	26.9 (23.5, 30.8)	33.2 (29.3, 37.6)	43.3 (38.0, 49.4)	55.2 (46.9, 65.0)
Model 1	Ref	1.15 (0.89, 1.50)	1.26 (0.99, 1.60)	1.64 (1.31, 2.05)	2.15 (1.72, 2.69)	Ref	0.97 (0.82, 1.14)	1.16 (0.99, 1.36)	1.43 (1.22, 1.68)	1.64 (1.35, 2.00)
Model 2	Ref	1.03 (0.79, 1.34)	0.98 (0.76, 1.25)	1.14 (0.90, 1.44)	1.38 (1.09, 1.75)	Ref	0.91 (0.77, 1.08)	0.99 (0.83, 1.16)	1.09 (0.92, 1.30)	1.23 (0.99, 1.54)
Model 3	Ref	1.01 (0.77, 1.31)	0.92 (0.72, 1.18)	0.93 (0.73, 1.18)	0.97 (0.75, 1.25)	Ref	0.90 (0.76, 1.07)	0.97 (0.82, 1.14)	1.01 (0.84, 1.20)	1.05 (0.83, 1.32)
Model 4	Ref	1.00 (0.77, 1.31)	0.92 (0.72, 1.18)	0.92 (0.72, 1.17)	0.96 (0.74, 1.25)	Ref	0.90 (0.76, 1.06)	0.96 (0.82, 1.14)	1.00 (0.83, 1.19)	1.03 (0.82, 1.30)
**Ischemic stroke**										
No. of events (%)	555 (0.3%)	766 (0.4%)	1519 (0.9%)	2545 (1.4%)	3464 (1.9%)	2037 (0.8%)	2203 (0.8%)	2704 (1.0%)	2617 (1.0%)	1361 (0.5%)
Incidence rate	386.6 (355.2, 420.6)	395.7 (367.8, 425.8)	462.2 (438.1, 487.6)	479.7 (459.7, 500.6)	614.9 (593.4, 637.1)	298.2 (284.8, 312.1)	348.1 (333.3, 363.6)	416.9 (400.5, 433.9)	535.8 (514.4, 558.0)	663.5 (628.1, 700.9)
Model 1	Ref	0.94 (0.85, 1.05)	1.08 (0.98, 1.19)	1.24 (1.13, 1.36)	1.65 (1.50, 1.80)	Ref	1.04 (0.98, 1.10)	1.15 (1.09, 1.22)	1.47 (1.39, 1.56)	1.87 (1.73, 2.01)
Model 2	Ref	0.94 (0.85, 1.05)	0.99 (0.89, 1.10)	1.02 (0.93, 1.13)	1.23 (1.11, 1.35)	Ref	1.09 (1.02, 1.16)	1.05 (0.98, 1.11)	1.04 (0.97, 1.11)	1.10 (1.01, 1.20)
Model 3	Ref	0.93 (0.84, 1.04)	0.96 (0.87, 1.06)	0.93 (0.84, 1.03)	1.05 (0.94, 1.17)	Ref	1.09 (1.02, 1.16)	1.04 (0.98, 1.11)	1.02 (0.96, 1.10)	1.06 (0.97, 1.15)
Model 4	Ref	0.93 (0.84, 1.04)	0.96 (0.87, 1.06)	0.94 (0.85, 1.03)	1.05 (0.94, 1.17)	Ref	1.08 (1.02, 1.15)	1.04 (0.98, 1.11)	1.02 (0.95, 1.09)	1.05 (0.96, 1.14)
**Hemorrhagic stroke**										
No. of events (%)	152 (0.1%)	228 (0.1%)	440 (0.2%)	785 (0.4%)	887 (0.5%)	527 (0.2%)	535 (0.2%)	554 (0.2%)	487 (0.2%)	260 (0.1%)
Incidence rate	106.4 (90.3, 125.4)	118.6 (103.5, 135.8)	134.4 (121.5, 148.8)	148.1 (137.0, 160.2)	156.0 (145.5, 167.3)	80.5 (73.7, 88.0)	87.2 (79.8, 95.3)	87.7 (80.3, 95.8)	101.9 (92.7, 112.0)	128.8 (113.5, 146.2)
Model 1	Ref	1.16 (0.94, 1.42)	1.31 (1.09, 1.57)	1.32 (1.11, 1.58)	1.35 (1.13, 1.61)	Ref	1.13 (1.00, 1.28)	1.15 (1.02, 1.30)	1.14 (1.01, 1.30)	1.09 (0.93, 1.28)
Model 2	Ref	1.01 (0.82, 1.24)	1.04 (0.86, 1.26)	0.96 (0.80, 1.15)	0.90 (0.75, 1.08)	Ref	1.04 (0.92, 1.17)	0.99 (0.87, 1.12)	0.94 (0.82, 1.08)	1.04 (0.87, 1.24)
Model 3	Ref	1.00 (0.82, 1.24)	1.03 (0.85, 1.24)	0.91 (0.75, 1.10)	0.82 (0.67, 1.01)	Ref	1.04 (0.92, 1.17)	0.98 (0.87, 1.12)	0.93 (0.81, 1.07)	1.02 (0.85, 1.22)
Model 4	Ref	1.01 (0.82, 1.24)	1.03 (0.85, 1.25)	0.91 (0.76, 1.10)	0.83 (0.68, 1.02)	Ref	1.04 (0.92, 1.17)	0.99 (0.87, 1.12)	0.94 (0.82, 1.07)	1.03 (0.86, 1.24)

Age-adjusted incidence rates were presented per 100,000 person-years.

Hazard ratios for developing cardiovascular events were calculated with Cox regression models.

Model 1 was stratified by age-at-risk (5-year group), and urban residency, and adjusted for BMI (continuous variable), systolic blood pressure (continuous variable), baseline status of diabetes (no/yes and treated/yes and untreated), baseline status of hypertension (no/yes and treated/yes and untreated), alcohol drinking (yes/no), metabolic equivalent of a day’s work and leisure activities (continuous variable), levels of education and income (each five categories), and the survey season (four categories).

Model 2 was stratified by age-at-risk (5-year group) and study areas (10 categories) instead of urban residency, and adjusted for BMI (continuous variable), systolic blood pressure (continuous variable), baseline diabetes (yes/no), alcohol drinking (yes/no), metabolic equivalent of a day’s work and leisure activities (continuous variable), levels of education and income (each five categories), and the survey season (four categories).

Model 3 was further adjusted for smoking-related variables, including smoking status (four categories), whether having smoked on the survey day (yes/no), and passive smoking.

Model 4 was further adjusted for solid fuel use-related variables, including solid fuel use for cooking (yes/no), solid fuel use for heating (yes/no), slow burning of solid fuel (yes/no) and ventilation at home (yes/no).

**Table 3:** The association between COex levels and future risk of CCVD by smoking status.

Outcomes	COex quintiles (ppm)									
	Men					Women				
	< 2.0	2.0–3.0	3.0–5.0	5.0–11.5	≥ 11.5	< 2.0	2.0–3.0	3.0–5.0	5.0–11.5	≥ 11.5
**Major coronary events**										
Never smokers										
No. of events (%)	34 (0.9%)	43 (0.9%)	98 (1.2%)	77 (1.2%)	20 (1.1%)	277 (0.4%)	243 (0.4%)	311 (0.5%)	293 (0.6%)	141 (0.8%)
Incidence rate	55.4 (37.9, 80.8)	58.4 (41.6, 82.0)	86.1 (66.7, 111.)	87.7 (67.5, 113.8)	87.4 (55.1, 138.7)	25.8 (22.3, 29.8)	25.8 (22.4, 29.7)	32.5 (28.5, 37.0)	42.6 (37.2, 48.9)	52.8 (44.1, 63.3)
HR (95% CI)	Ref	0.89 (0.56, 1.42)	1.04 (0.68, 1.59)	0.87 (0.55, 1.37)	0.87 0.46, 1.62)	Ref	0.92 (0.77, 1.10)	1.01 (0.85, 1.20)	1.08 (0.89, 1.30)	1.09 (0.85, 1.40)
Current regular smokers										
No. of events (%)	16 (0.6%)	27 (0.7%)	71 (0.8%)	316 (1.0%)	657 (1.0%)	9 (2.5%)	10 (2.4%)	13 (2.0%)	28 (1.5%)	35 (1.6%)
Incidence rate	45.2 (27.7, 73.8)	55.1 (37.6, 80.8)	62.0 (48.9, 78.6)	80.3 (70.8, 91.0)	112.0(102.3, 122.6)	123.1 (58.7, 258.4)	136.7 (66.6, 280.8)	123.7 (68.1, 224.7)	104.4 (64.3, 169.6)	156.0 (102.8, 236.7)
HR (95% CI)	Ref	1.03 (0.55, 1.92)	0.98 (0.57, 1.70)	0.98 (0.58, 1.65)	1.06 (0.63, 1.80)	Ref	0.68 (0.26, 1.79)	0.69 (0.27, 1.78)	0.37 (0.15, 0.91)	0.5 (0.19, 1.28)
**Ischemic stroke**										
Never smokers										
No. of events (%)	220 (5.7%)	266 (5.4%)	469 (5.6%)	411 (6.2%)	134 (7.4%)	1970 (3.1%)	2102 (3.3%)	2579 (4.0%)	2380 (5.1%)	1138 (6.2%)
Incidence rate	483.4 (420.8, 555.4)	475.5 (418.0, 540.9)	538.7 (486.3, 596.7)	615.3 (552.8, 684.8)	765.1 (644.3, 908.6)	292.9 (279.6, 306.9)	338.6 (323.8, 354.1)	409.7 (393.2, 426.8)	527.4 (505.5, 550.3)	645.0 (607.6, 684.6)
HR (95% CI)	Ref	0.95 (0.79, 1.15)	0.96 (0.81, 1.15)	0.95 (0.79, 1.15)	1.03 (0.80, 1.33)	Ref	1.08 (1.02, 1.15)	1.05 (0.98, 1.12)	1.02 (0.95, 1.10)	1.02 (0.93, 1.12)
Current regular smokers										
No. of events (%)	65 (2.6%)	127 (3.4%)	333 (3.7%)	1372 (4.3%)	3042 (4.7%)	21 (5.8%)	29 (6.9%)	35 (5.3%)	139 (7.6%)	189 (8.6%)
Incidence rate	214.3 (168.1, 273.2)	297.9 (250.4, 354.3)	334.9 (300.2, 373.5)	401.0 (378.6, 424.8)	573.5 (551.6, 596.4)	533.4 (342.8, 829.9)	686.4 (475.8, 990.4)	551.5 (391.1, 777.7)	846.6 (709.1, 1011.0)	1209.0 (1045.0, 1399.0)
HR (95% CI)	Ref	1.32 (0.98, 1.78)	1.26 (0.96, 1.65)	1.27 (0.98, 1.64)	1.37 (1.06, 1.77)	Ref	1.00 (0.55, 1.81)	0.76 (0.42, 1.36)	0.93 (0.55, 1.57)	1.12 (0.65, 1.93)
**Hemorrhagic stroke**										
Never smokers										
No. of events (%)	47 (1.2%)	66 (1.3%)	128 (1.5%)	95 (1.4%)	38 (2.1%)	486 (0.8%)	510 (0.8%)	520 (0.8%)	433 (0.9%)	233 (1.3%)
Incidence rate	113.2 (83.7, 153.2)	128.4 (99.5, 165.8)	159.1 (130.6, 193.7)	152.9 (123.5, 189.2)	230.7 (165.6, 321.3)	76.4 (69.6, 83.9)	85.8 (78.3, 93.9)	85.8 (78.3, 93.9)	99.0 (89.6, 109.3)	135.6 (118.8, 154.8)
HR (95% CI)	Ref	0.96 (0.66, 1.41)	1.06 (0.74, 1.51)	0.87 (0.59, 1.27)	1.20 (0.74, 1.96)	Ref	1.08 (0.95, 1.23)	1.02 (0.89, 1.16)	0.94 (0.81, 1.09)	1.09 (0.90, 1.32)
Current regular smokers										
No. of events (%)	41 (1.7%)	59 (1.6%)	135 (1.5%)	497 (1.6%)	785 (1.2%)	10 (2.8%)	7 (1.7%)	12 (1.8%)	30 (1.6%)	22 (1 .0%)
Incidence rate	127.5 (93.5, 173.9)	131.1 (101.1, 170.1)	128.1 (107.7, 152.4)	136.8 (123.7, 151.3)	140.9 (130.3, 152.3)	284.1 (150.7, 535.5)	178.4 (86.4, 368.7)	205.5 (117.2, 360.3)	192.1 (128.3, 287.6)	140.7 (91.7, 215.9)
HR (95% CI)	Ref	0.95 (0.63, 1.41)	0.88 (0.62, 1.26)	0.79 (0.57, 1.11)	0.68 (0.49, 0.96)	Ref	0.57 (0.20, 1.64)	0.79 (0.31, 2.03)	0.72 (0.30, 1.72)	0.51 (0.19, 1.35)

Age-adjusted incidence rates were presented per 100,000 person-years.

Hazard ratios for developing cardiovascular events were calculated with Cox regression models stratified by age-at-risk (5-year group) and study areas (10 categories), and adjusted for BMI (continuous variable), systolic blood pressure (continuous variable), baseline status of diabetes (no/yes and treated/yes and untreated), baseline status of hypertension (no/yes and treated/yes and untreated), alcohol drinking (yes/no), metabolic equivalent of a day’s work and leisure activities (continuous variable), levels of education and income (each five categories), the survey season (four categories), smoking-related variables including passive smoking (yes/no), plus whether having smoked on the survey day (yes/no), cigarette equivalents/day, and depth of inhalation (mouth /throat/lung) among current regular smokers, and solid fuel use-related variables including solid fuel use for cooking (yes/no), solid fuel use for heating (yes/no), slow burning of solid fuel (yes/no) and ventilation at home (yes/no).

The number of cigarette equivalents smoked per day was calculated for current and ex-regular smokers including all types of tobacco, with one pipe or one hand-rolled cigarette being treated as equal to 5/3 cigarettes, and one cigar being treated as equal to 2 cigarettes.

**Figure 1:**
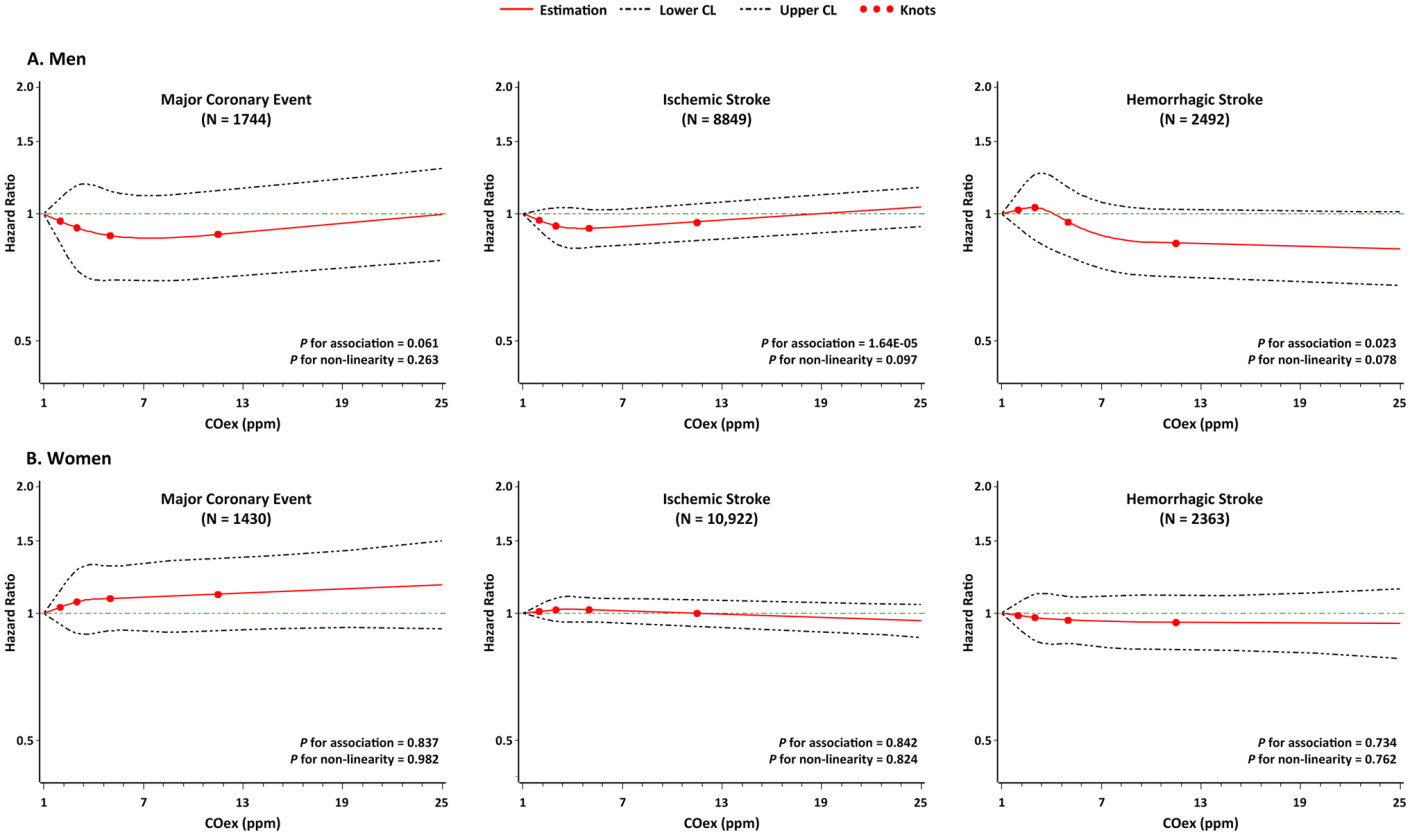
Restricted cubic splines displaying the association of COex levels with future risk of CCVD. Cox models were adjusted for age-at-risk (5-year group), study areas (10 categories), BMI (continuous variable), systolic blood pressure (continuous variable), baseline status of diabetes (no/yes and treated/yes and untreated), baseline status of hypertension (no/yes and treated/yes and untreated), alcohol drinking (yes/no), metabolic equivalent of a day’s work and leisure activities (continuous variable), levels of education and income (each five categories), and the survey season (four categories), whether smoked on the survey day (yes/no), and smoking status (four categories), passive smoking (yes/no), solid fuel use for cooking (yes/no), solid fuel use for heating (yes/no), slow burning of solid fuel (yes/no) and ventilation at home (yes/no); Red lines show the association of exhaled CO with the risk of future major cardiovascular disease; upper and lower 95 confidence limits are plotted as black dashed lines, with knots corresponding to COex quintiles. This graph was plotted with SAS 9.4 using the “%lgtphcurv9” macro (https://www.hsph.harvard.edu/donna-spiegelman/software/lgtphcurv9/).

**Figure 2:**
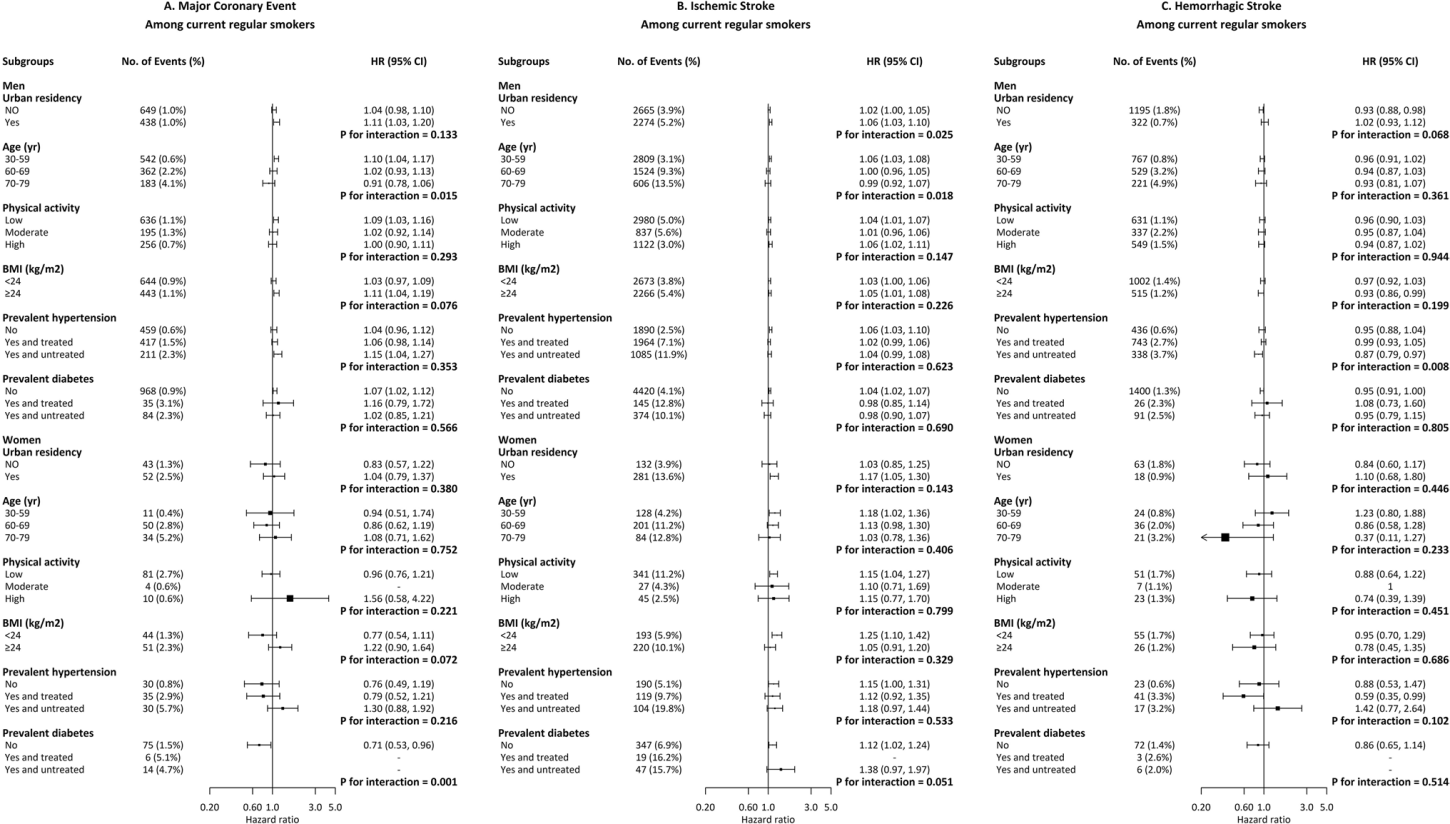
The association of COex levels with future risk of CCVD according to subgroups of baseline characteristics among current regular smokers. Physical activity level was categorized according to guidelines for data processing and analysis of the international physical activity questionnaire (IPAQ)-short and long forms (https://www.ipaq.ki.se/scoring.pdf). High physical activity was defined as vigorous-intensity activity achieving a total physical activity of at least 1500 MET-minutes/week or any combination of walking, moderate-intensity or vigorous-intensity activities achieving a total physical activity of at least 3000 MET-minutes/week; Moderate physical activity was defined as any combination of walking, moderate-intensity or vigorous intensity activities achieving a total physical activity of at least 600 MET-minutes/week; Low physical activity was defined if not meeting criteria for “High” or “Moderate”. Hazard ratios were presented per 7 ppm (interquartile range) increase in COex levels. Cox models was stratified by age-at-risk (5-year group) and study areas (10 categories), and adjusted for BMI (continuous variable), systolic blood pressure (continuous variable), baseline status of diabetes (no/yes and treated/yes and untreated), baseline status of hypertension (no/yes and treated/yes and untreated), alcohol drinking (yes/no), metabolic equivalent of a day’s work and leisure activities (continuous variable), levels of education and income (each five categories), the survey season (four categories), whether smoked on the survey day (yes/no), cigarette equivalents/day (continuous variable), and depth of inhalation (mouth/throat/lung), passive smoking (yes/no), solid fuel use for cooking (yes/no), solid fuel use for heating (yes/no), slow burning of solid fuel (yes/no) and ventilation at home (yes/no). This graph was plotted with R 3.6.0 using the “forestplot” package (https://cran.r-project.org/web/packages/forestplot/index.html).

In the RCS plots (Fig. 1 and Supplementary Figs. S4–S5), a slight but significant trend of increasing risk for ischemic stroke (*P* for association = 1.64E-05) was revealed after full adjustment among men in association with higher COex level, which was more pronounced among male smokers (*P* for association = 0.017), and such association was also emerged among female smokers (*P* for association = 0.047). The meta-analysis of the associations across 10 study areas revealed similar associations (Supplementary Figs. S6–S8), and no major heterogeneity was found across different areas (*P* for heterogeneity all > 0.05). In further subgroup analysis by urban residency and cardiovascular risk factors (Fig. 2 and Supplementary Fig. S9), we found that the associations with major coronary events and ischemic stroke were occasionally observed among male smokers, which were more pronounced among those who were under 60 years of age (*P*_interaction_ = 0.015 and 0.018, respectively). We also observed a positive association with major coronary events among female smokers with prevalent diabetes (*P*_interaction_ = 0.001), however, with doubtable validity considering the small number of outcome cases in this subgroup (N = 20).

In the sensitivity analyses excluding events occurring within the first two years of follow-up, the results remained essentially unchanged (Supplementary Table S2). When examining fatal CCVD events (Supplementary Table S3), we also observed that the association of COex with total CCVD death and fatal ischemic event was positive before adjusting for study areas, smoking and solid fuel use (HR [95% CI]: 1.07 [0.91, 1.26], *P*_trend_ =  0.049  and 1.18 [0.91, 1.54], *P*_trend_ =  0.015  among men), and null after such adjustment.

## Discussion

In this large prospective study of Chinese adults, we observed positive associations of COex level with cigarette consumption and household solid fuel use, and suggestively, with ambient CO levels, supporting the notion that COex could at least crudely reflect the integrated level of CO exposure. We then found that higher COex level was associated with increased risk of major coronary events and ischemic stroke, which was not independent of sources of CO exposure, although independent association seemed to exist within the subgroup of male smokers, while the positive association with hemorrhagic was less strong and susceptible to adjustment of CO exposure.

Our finding that higher COex level was associated with increased risk of ischemic CCVD, but not independently of CO exposure, was similar to that revealed in previous studies in that, the association was attenuated by adjustment of variables related with CO exposure, while there existed discrepancy regarding the extent of the attenuation. The Framingham study found that elevated COex levels was associated with increased risk of future cardiovascular events (416 cases), risk of stroke/transient ischemic attack (231 cases), and risk of progressing from subclinical to clinical CVD (193 cases), independently of smoking status^11–13^. However, these studies did not take into account of the dose of cigarette smoking for adjustment, and was conducted within a single geographic location, thus limiting the generalizability of the study findings. When cigarette equivalent dose was adjusted for, the Scottish Heart Health Extended Cohort study, which included participants from various districts across Scotland, found that positive association between COex level and risk of CHD (3098 cases) and PAD (499 cases) were attenuated but maintained whereas the association with AF (1036 cases) risk was abolished^14,15^. There was another study which recruited participants from an urban, a semi-urban and a rural area across France, and used stratified Cox regression analysis by study locations, similar to that in our study, and this study also revealed a similar finding to ours that the positive association between COex levels and CVD mortality (37 cases) was attenuated to the null after adjustment for smoking (including smoking status and pack-years)^16^. It could be conjured that the discrepancy within these studies may result from differences in adjustment for CO exposure variables. In the present study, we performed comprehensive adjustment for CO exposure, including intensity and depth of smoking, household solid fuel use, and study areas as a proxy for ambient CO exposure, and found that COex level was associated with increased risk of major coronary events and ischemic stroke, which was not independent of CO exposure. This finding suggested that, though not being an independent CVD risk factor, COex could potentially serve as a cost-effective biomarker for ischemic cardiovascular risk when exposed to CO.

Notably, the independent association between COex level and ischemic cardio-cerebral-vascular risk was not completely absent in our study. It appeared that COex level remained to be associated with increased risk of ischemic stroke among male smokers after adjustment for proxy variables of CO exposure, and the associated risk was decreasing with increasing age, which was consistent with the observation that older individuals exhibited reduced responsiveness to sympathetic and autonomic nervous system stimuli, while sympathetic activation is considered as a manifestation of early cardiovascular injuries^21^. We also observed that the association between COex level and ischemic stroke within male smokers was more pronounced among those living in urban areas. In Chinese cities, ambient CO and PM_2.5_ was moderately correlated with correlation coefficients around 0.6^22–24^, as revealed in previous time-series studies. By contrast, in rural China where solid fuel use constituted a source of CO pollution, the correlation between CO and PM_2.5_ in emissions from solid fuel combustion varied greatly, with correlation coefficients ranging from 0.10 to 0.96^25^. Considering that PM_2.5_ exposure has been established as an independent cardiovascular risk factor^26^, we conjectured that the association between COex level and ischemic stroke observed among urban smokers might be partly owing to inadequate adjustment for exposure of PM_2.5_, which was correlated with CO, or depending on certain interactions between CO and PM_2.5_.

The relation of COex with hemorrhagic stroke was less clear in our study. A weak positive association was found in the whole population without adjustment for proxy variables of CO exposure; however, after this adjustment, a negative association was observed among current regular smokers. Previous time-series studies on ambient CO levels and hemorrhagic stroke were controversial, with reports of positive associations^27,28^, as well as null associations negative in direction^29,30^, while evidence from murine models suggested that circulating CO, namely carboxyhemoglobin, was protective against hemorrhagic shock by delaying reperfusion injury during resuscitation^31,32^. Our finding was also far from being conclusive, that the association of COex with hemorrhagic stroke was susceptible to adjustment for CO exposure. The true association between CO exposure and hemorrhagic stroke remains to be revealed by future studies from other populations.

The primary strength of our study lies in its prospective design and a large study sample from 10 diverse study areas across China. To the best of our knowledge, this is the largest study so far examining association between COex and cardiovascular risk, and the first one conducted in the Chinese population. Besides, we examined the association of COex level with various cardiovascular endpoints individually, including hemorrhagic stroke which had not been investigated in previous studies. In addition, we provided evidence that COex level was associated with various sources of CO exposure and performed comprehensive adjustment for such sources, including intensity and depth of smoking and household solid fuel use, which were not available in previous studies.

Our study also has several limitations. First, measurements of COex level was only available at baseline for all participants, and we used one baseline measurement to explore the association between COex level and future CCVD risk. It was reassuring, however, that changes in exposure would generally dilute rather than overestimate the associated risk^33,34^. Second, we did not obtain and were not able to adjust for baseline ambient CO levels at the residential locations of the participants. Nevertheless, we performed stratified adjustment of study areas in our analysis, and the exposure level to ambient CO was believed to be similar among participants from the same study area. Third, although we performed extensive adjustment for traditional cardiovascular risk factors in this study, residual confounding from unavailable CCVD risk factors such as blood lipid profile remains possible. Finally, our study population consisted mainly of middle-to-elderly aged Chinese adults; as a result, caution must be taken when generalizing our findings to other populations.

## Conclusion

Higher COex level (over 3 ppm) was associated with elevated risk of ischemic CCVD, but not independently of CO exposure, although an independent association seemed to exist within male smokers. Our study suggests that, not being an independent cardiovascular risk factor in general, COex level could potentially serve as a cost-effective biomarker for ischemic cardiovascular risk, given that CO exposure is ubiquitous.

## Supplementary information

Supplementary Information.
