# Supplementary material for: Association of exhaled carbon monoxide with risk of cardio-cerebral-vascular disease in the China Kadoorie Biobank cohort study
Source: Sci Rep. 2020 Nov 11;10:19507. doi: 10.1038/s41598-020-76353-2 (PMC7659340; doi:10.1038/s41598-020-76353-2)
Supplement: Supplementary file 1 — Supplementary Information. [file 41598_2020_76353_MOESM1_ESM.docx]

**Association of exhaled carbon monoxide with risk of cardio-cerebral-vascular disease in the China Kadoorie Biobank cohort study**

**Running title: Exhaled CO and cardio-cerebral-vascular disease**

Gaokun Qiu, Kuai Yu, Canqing Yu, Wending Li, Jun Lv, Yu Guo, Zheng Bian, Ling Yang, Yiping Chen, Zhengming Chen, Frank B. Hu, Liming Li and Tangchun Wu on behalf of the China Kadoorie Biobank Collaborative Group

**List of Study Committees and Investigators in the China Kadoorie Biobank Collaborative Group**

**International Steering Committee:** Junshi Chen, Zhengming Chen (PI), Rory Collins, Liming Li (PI), Richard Peto.

**International Co-ordinating Centre, Oxford:** Daniel Avery, Derrick Bennett, Yumei Chang, Yiping Chen, Zhengming Chen, Robert Clarke, Huaidong Du, Xuejuan Fan, Simon Gilbert, Alex Hacker, Michael Holmes, Andri Iona, Christiana Kartsonaki; Rene Kerosi, Ling Kong, Om Kurmi, Garry Lancaster, Sarah Lewington, John McDonnell, Winnie Mei, Iona Millwood, Qunhua Nie, Jayakrishnan Radhakrishnan, Sajjad Rafiq, Paul Ryder, Sam Sansome, Dan Schmidt, Paul Sherliker, Rajani Sohoni, Iain Turnbull, Robin Walters, Jenny Wang, Lin Wang, Ling Yang, Xiaoming Yang. **National Co-ordinating Centre, Beijing:** Zheng Bian, Ge Chen, Yu Guo, Bingyang Han, Can Hou, Jun Lv, Pei Pei, Shuzhen Qu, Yunlong Tan, Canqing Yu, Huiyan Zhou. **10 Regional Co-ordinating Centres: Qingdao** Qingdao CDC: Zengchang Pang, Ruqin Gao, Shaojie Wang, Yongmei Liu, Ranran Du, Yajing Zang, Liang Cheng, Xiaocao Tian, Hua Zhang. Licang CDC: Silu Lv, Junzheng Wang, Wei Hou. **Heilongjiang** Provincial CDC: Jiyuan Yin, Ge Jiang, Shumei Liu, Zhigang Pang, Xue Zhou. Nangang CDC: Liqiu Yang, Hui He, Bo Yu, Yanjie Li, Huaiyi Mu, Qinai Xu, Meiling Dou, Jiaojiao Ren. **Hainan** Provincial CDC: Jianwei Du, Shanqing Wang, Ximin Hu, Hongmei Wang, Jinyan Chen, Yan Fu, Zhenwang Fu, Xiaohuan Wang, Hua Dong. Meilan CDC: Min Weng, Xiangyang Zheng, Yijun Li, Huimei Li, Chenglong Li. **Jiangsu** Provincial CDC: Ming Wu, Jinyi Zhou, Ran Tao, Jie Yang. Suzhou CDC: Jie Shen, Yihe Hu, Yan Lu, Yan Gao, Liangcai Ma, Renxian Zhou, Aiyu Tang, Shuo Zhang, Jianrong Jin. **Guangxi** Provincial CDC: Zhenzhu Tang, Naying Chen, Ying Huang. Liuzhou CDC: Mingqiang Li, Jinhuai Meng, Rong Pan, Qilian Jiang, Jingxin Qing, Weiyuan Zhang, Yun Liu, Liuping Wei, Liyuan Zhou, Ningyu Chen, Jun Yang, Hairong Guan. **Sichuan** Provincial CDC: Xianping Wu, Ningmei Zhang, Xiaofang Chen, Xuefeng Tang. Pengzhou CDC: Guojin Luo, Jianguo Li, Xiaofang Chen, Jian Wang, Jiaqiu Liu, Qiang Sun. **Gansu** Provincial CDC: Pengfei Ge, Xiaolan Ren, Caixia Dong. Maiji CDC: Hui Zhang, Enke Mao, Xiaoping Wang, Tao Wang. **Henan** Provincial CDC: Guohua Liu, Baoyu Zhu, Gang Zhou, Shixian Feng, Liang Chang, Lei Fan. Huixian CDC: Yulian Gao, Tianyou He, Li Jiang, Huarong Sun, Pan He, Chen Hu, Qiannan Lv, Xukui Zhang. **Zhejiang** Provincial CDC: Min Yu, Ruying Hu, Le Fang, Hao Wang. Tongxiang CDC: Yijian Qian, Chunmei Wang, Kaixue Xie, Lingli Chen, Yaxing Pan, Dongxia Pan. **Hunan** Provincial CDC: Yuelong Huang, Biyun Chen, Donghui Jin, Huilin Liu, Zhongxi Fu, Qiaohua Xu. Liuyang CDC: Xin Xu, Youping Xiong, Weifang Jia, Xianzhi Li, Libo Zhang, Zhe Qiu.


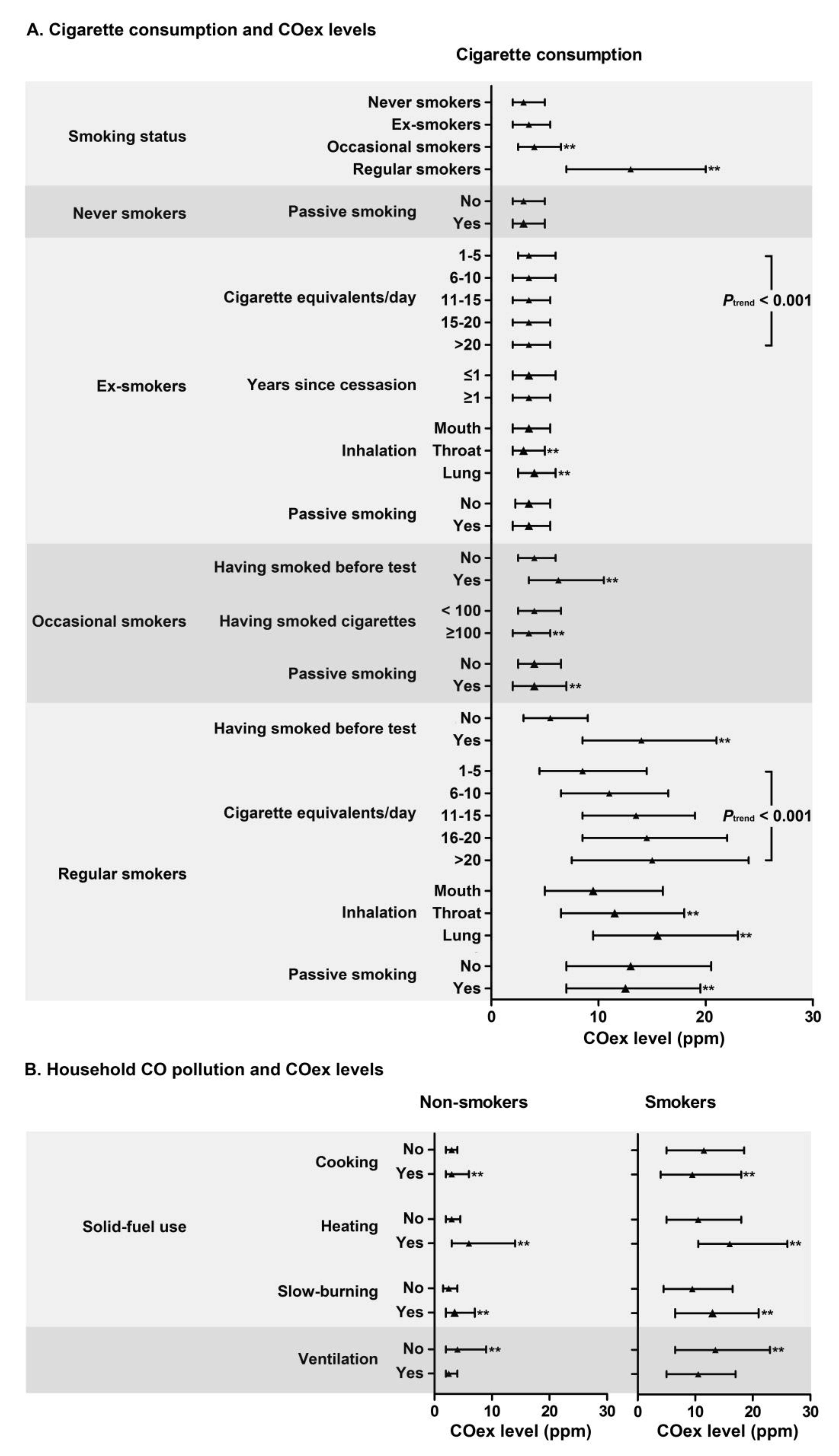


Supplementary Figure S1. The associations between cigarette consumption and household CO pollution with COex levels. The horizontal lines showed COex levels with interquartile ranges.

^*^*P* < 0.05; ^**^*P* < 0.01. *P* values obtained from linear regression models adjusted for age, sex, and urban residency, with COex logarithmically transformed.


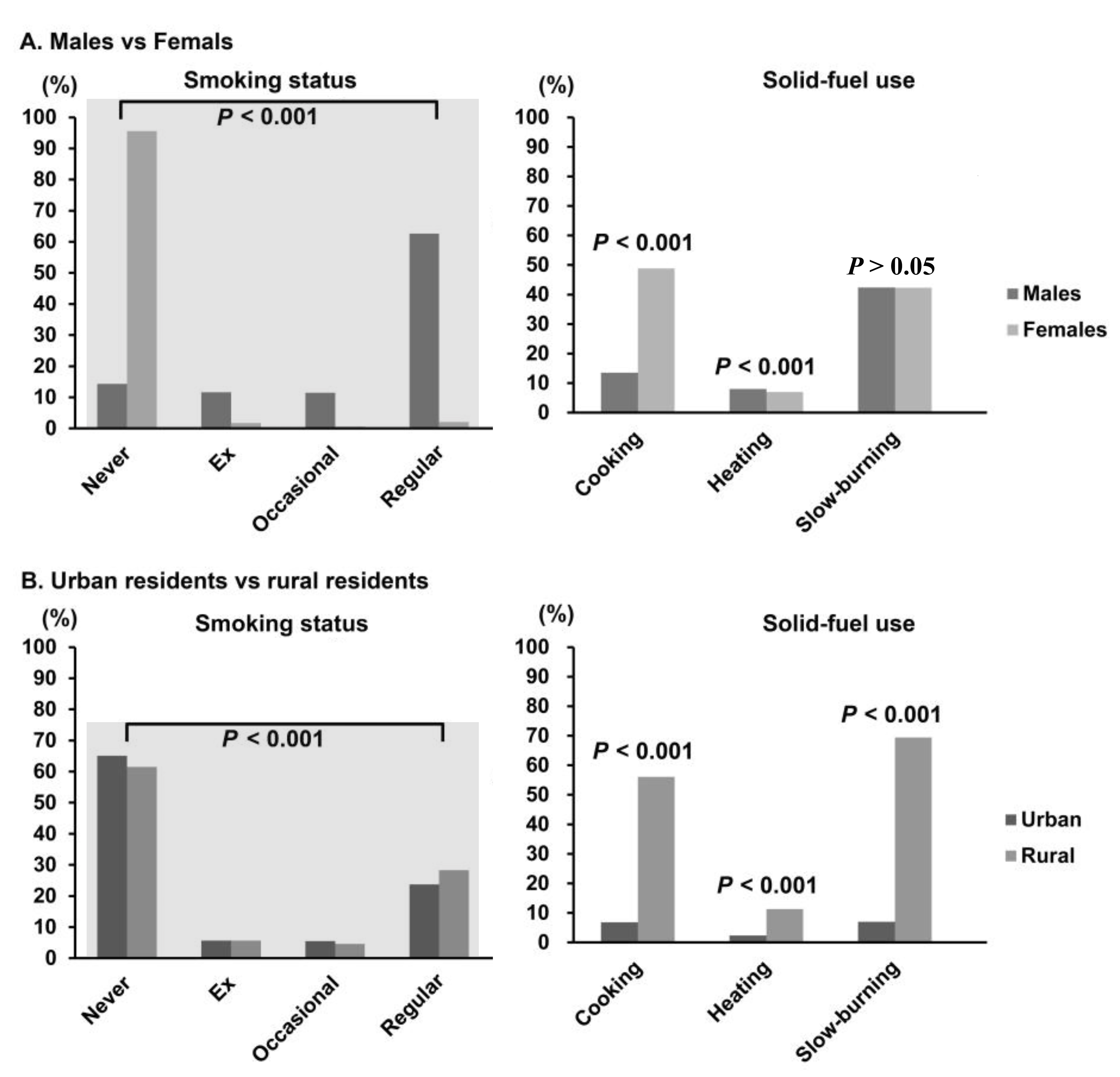


Supplementary Figure S2. Differences in CO exposure pattern between males and females (A), and urban and rural residents (B). The vertical bars showed participants who were never/ex/occasional/regular smokers among males and females (A), and among rural and urban residents (B), and of those who reported to use solid fuels for cooking, for heating, or for slow-burning among males and females (A), and among rural and urban residents (B), respectively.

*P* values were obtained with Chi-square tests.


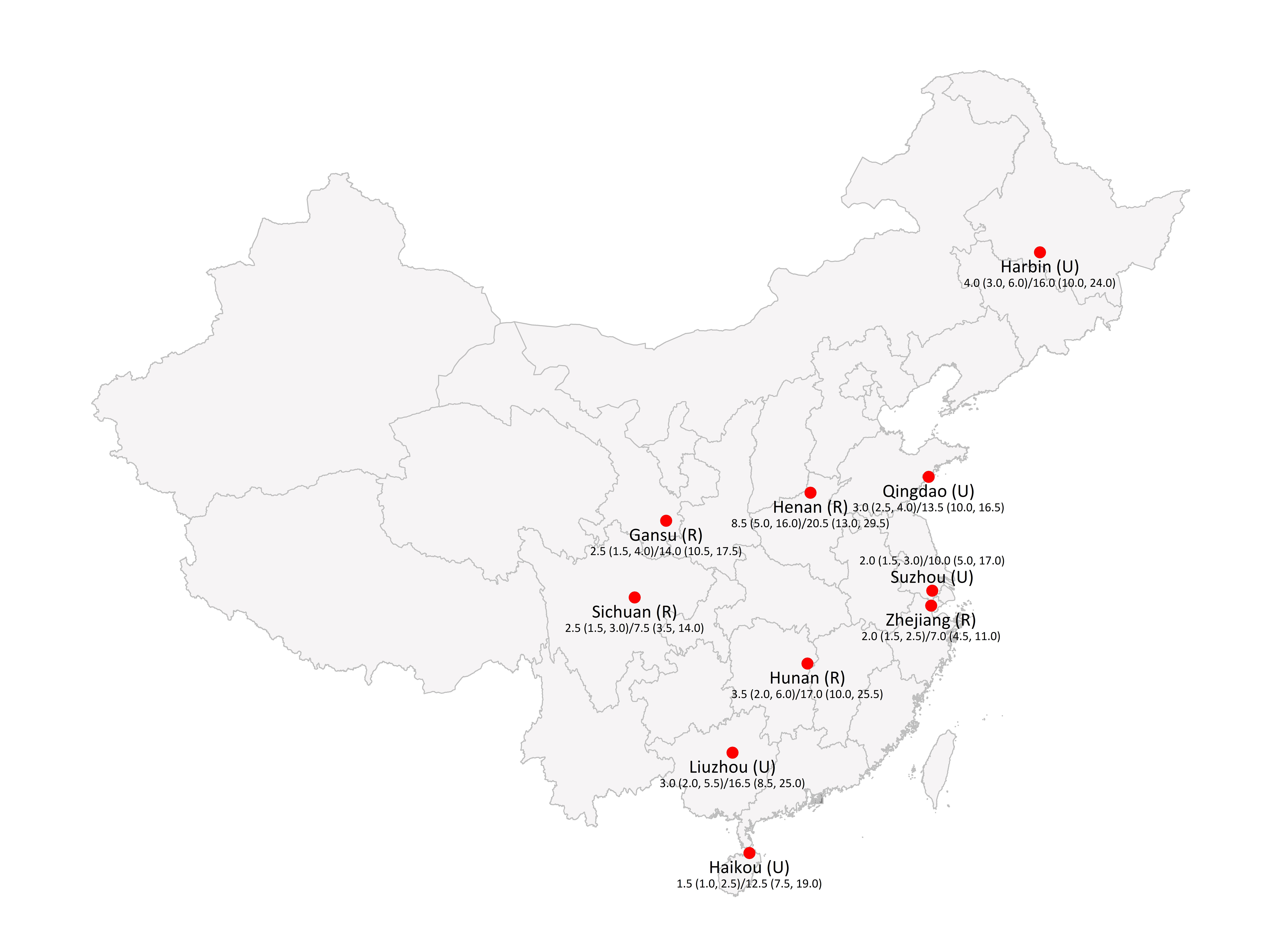


Supplementary Figure S3. COex levels among never smokers (before slash) and current regular smokers (after slash) across the 10 study areas in mainland China. Medians with interquartile ranges of COex were provided. This map was plotted with R 3.6.0 using packages “ggplot2” (the tool for data visualization, https://cran.r-project.org/web/packages/maptools/index.html), “rgeos” (the interface to geometry engine, https://cran.r-project.org/web/packages/rgeos/index.html) and “maptools” (the tool for handling spatial objects, https://cran.r-project.org/web/packages/maptools/index.html).


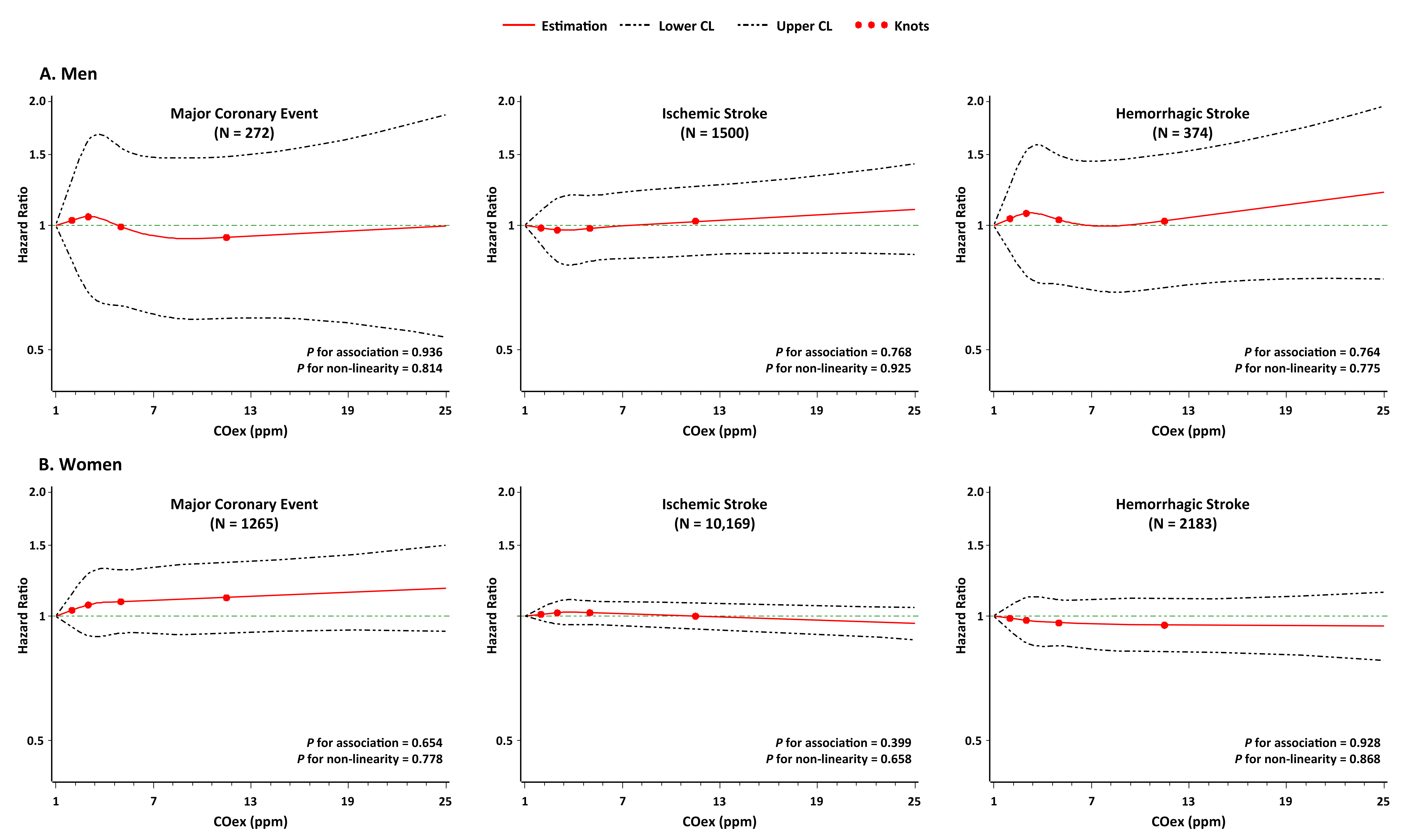


Supplementary Figure S4. Restricted cubic splines displaying the association of COex levels with future risk of major cardiovascular disease among never smokers.

Cox models were adjusted for age-at-risk (5-year group), study areas (10 categories), BMI, systolic blood pressure, baseline status of diabetes (no/yes and treated/yes and untreated), baseline status of hypertension (no/yes and treated/yes and untreated), alcohol drinking, metabolic equivalent of a day’s work and leisure activities, levels of education and income, the survey season, passive smoking, solid fuel use for cooking, solid fuel use for heating, slow burning of solid fuel and ventilation at home (yes/no);

Red lines show the association of exhaled CO with the risk of future major cardiovascular disease; upper and lower 95 confidence limits are plotted as black dashed lines, with knots corresponding to COex quintiles. This graph was plotted with SAS 9.4 using the “%lgtphcurv9” macro (https://www.hsph.harvard.edu/donna-spiegelman/software/lgtphcurv9/).


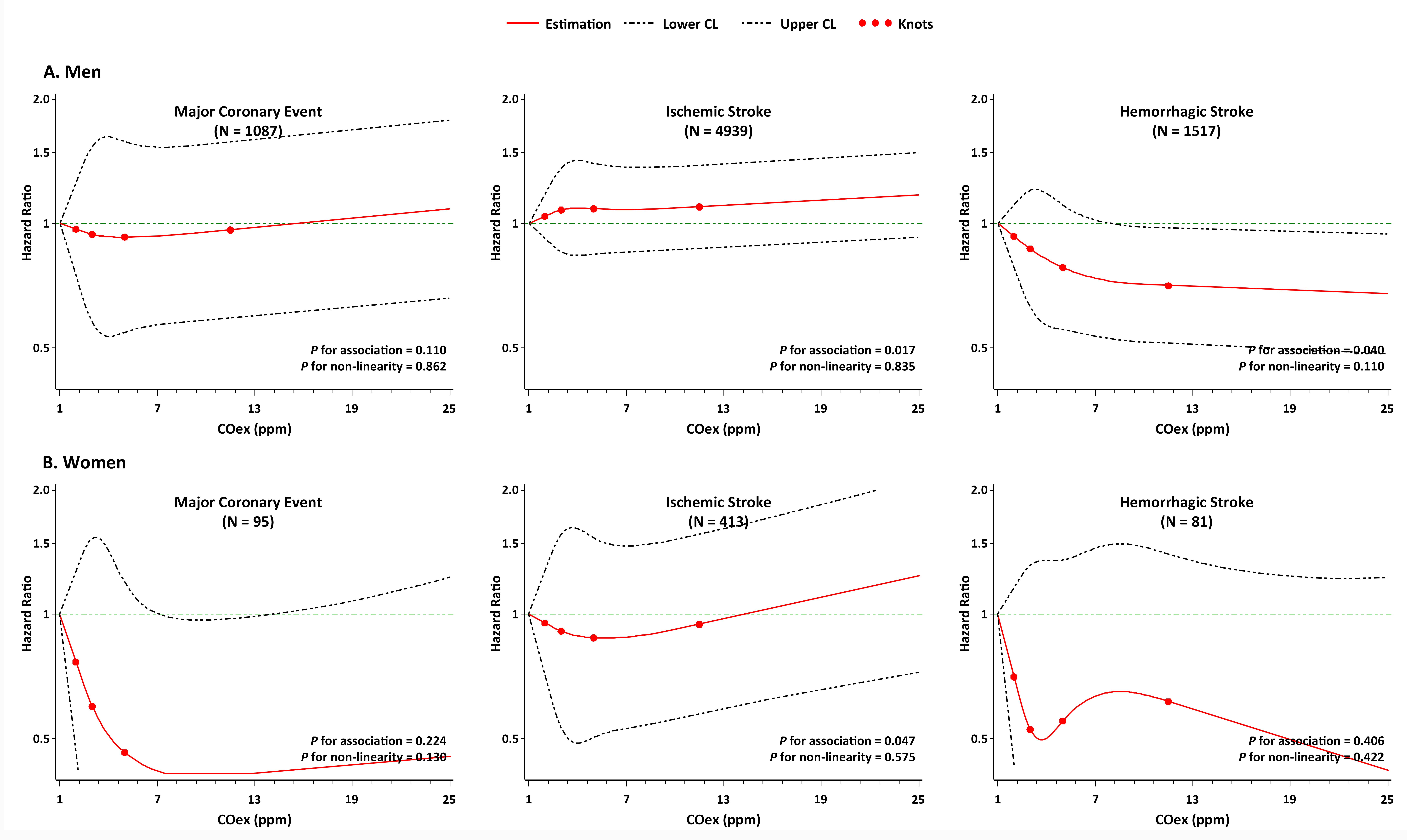


Supplementary Figure S5. Restricted cubic splines displaying the association of COex levels with future risk of major cardiovascular disease among current regular smokers.

Cox models were adjusted for age-at-risk (5-year group), study areas (10 categories), BMI, systolic blood pressure, baseline status of diabetes (no/yes and treated/yes and untreated), baseline status of hypertension (no/yes and treated/yes and untreated), alcohol drinking, metabolic equivalent of a day’s work and leisure activities, levels of education and income, the survey season, whether smoked on the survey day, cigarette equivalents/day, and depth of inhalation, passive smoking, solid fuel use for cooking, solid fuel use for heating, slow burning of solid fuel and ventilation at home (yes/no);

Red lines show the association of exhaled CO with the risk of future major cardiovascular disease; upper and lower 95 confidence limits are plotted as black dashed lines, with knots corresponding to COex quintiles. This graph was plotted with SAS 9.4 using the “%lgtphcurv9” macro (https://www.hsph.harvard.edu/donna-spiegelman/software/lgtphcurv9/).


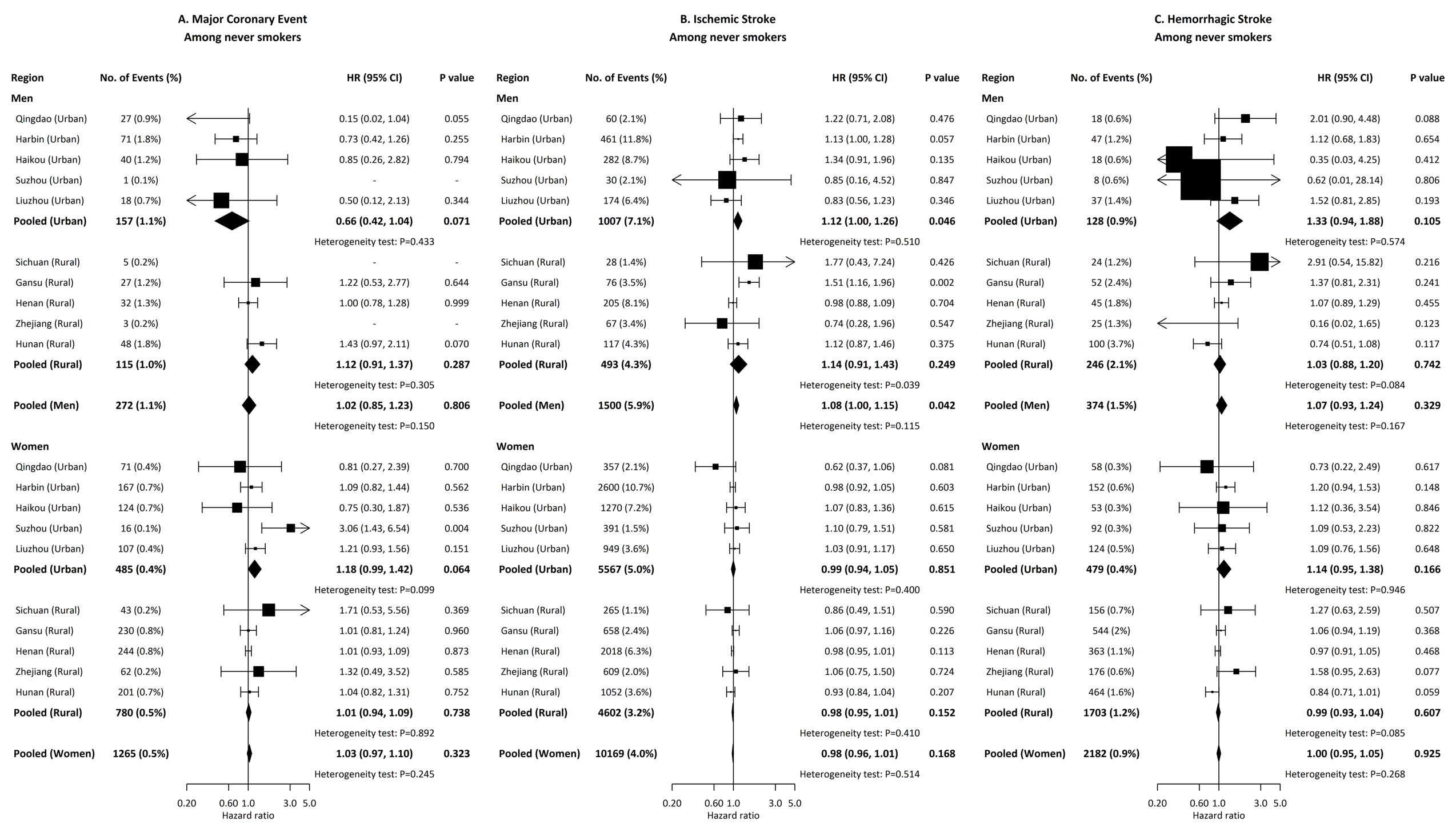


Supplementary Figure 6. The association of COex levels with future risk of major cardiovascular events across 10 study regions among never smokers. The black boxes represent hazard ratios, with the size inversely proportional to the variance of the logarithm of the hazard ratio.

Hazard ratios were presented per 7 ppm (interquartile range) increase in COex levels; Cox models were stratified by age-at-risk (5-year group), and adjusted for BMI, systolic blood pressure, baseline status of diabetes (no/yes and treated/yes and untreated), baseline status of hypertension (no/yes and treated/yes and untreated), alcohol drinking, metabolic equivalent of a day’s work and leisure activities, levels of education and income, the survey season, passive smoking, solid fuel use for cooking, solid fuel use for heating, slow burning of solid fuel and ventilation at home (yes/no). This graph was plotted with R 3.6.0 using the “forestplot” package (https://cran.r-project.org/web/packages/forestplot/index.html).


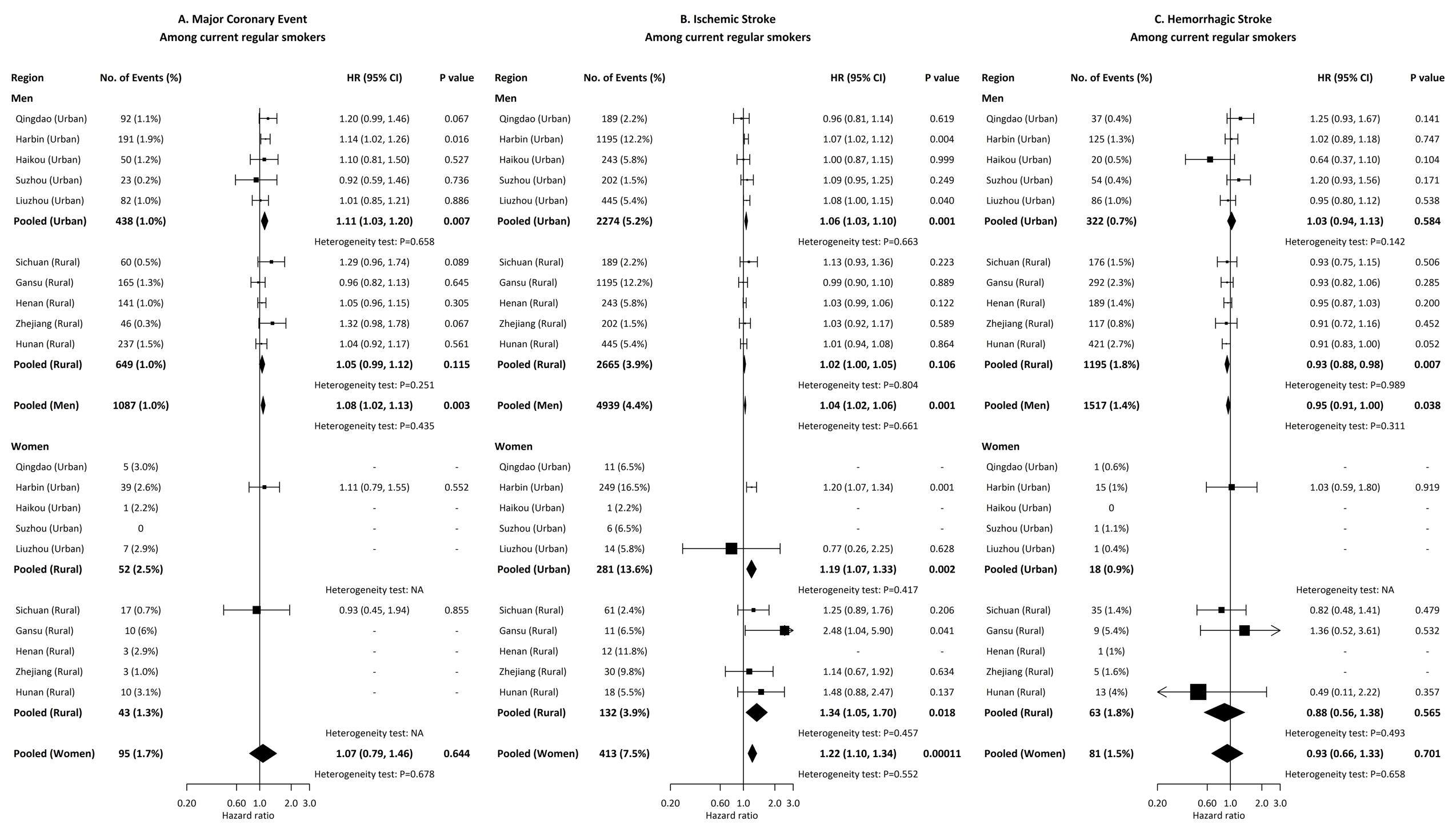


Supplementary Figure S7 The association of COex levels with future risk of major cardiovascular events across 10 study regions among current regular smokers. The black boxes represent hazard ratios, with the size inversely proportional to the variance of the logarithm of the hazard ratio.

Hazard ratios were presented per 7 ppm (interquartile range) increase in COex levels; Cox models were stratified by age-at-risk (5-year group), and adjusted for BMI, systolic blood pressure, baseline status of diabetes (no/yes and treated/yes and untreated), baseline status of hypertension (no/yes and treated/yes and untreated), alcohol drinking, metabolic equivalent of a day’s work and leisure activities, levels of education and income, the survey season, whether smoked on the survey day, cigarette equivalents/day, and depth of inhalation, passive smoking, solid fuel use for cooking, solid fuel use for heating, slow burning of solid fuel and ventilation at home. This graph was plotted with R 3.6.0 using the “forestplot” package (https://cran.r-project.org/web/packages/forestplot/index.html).


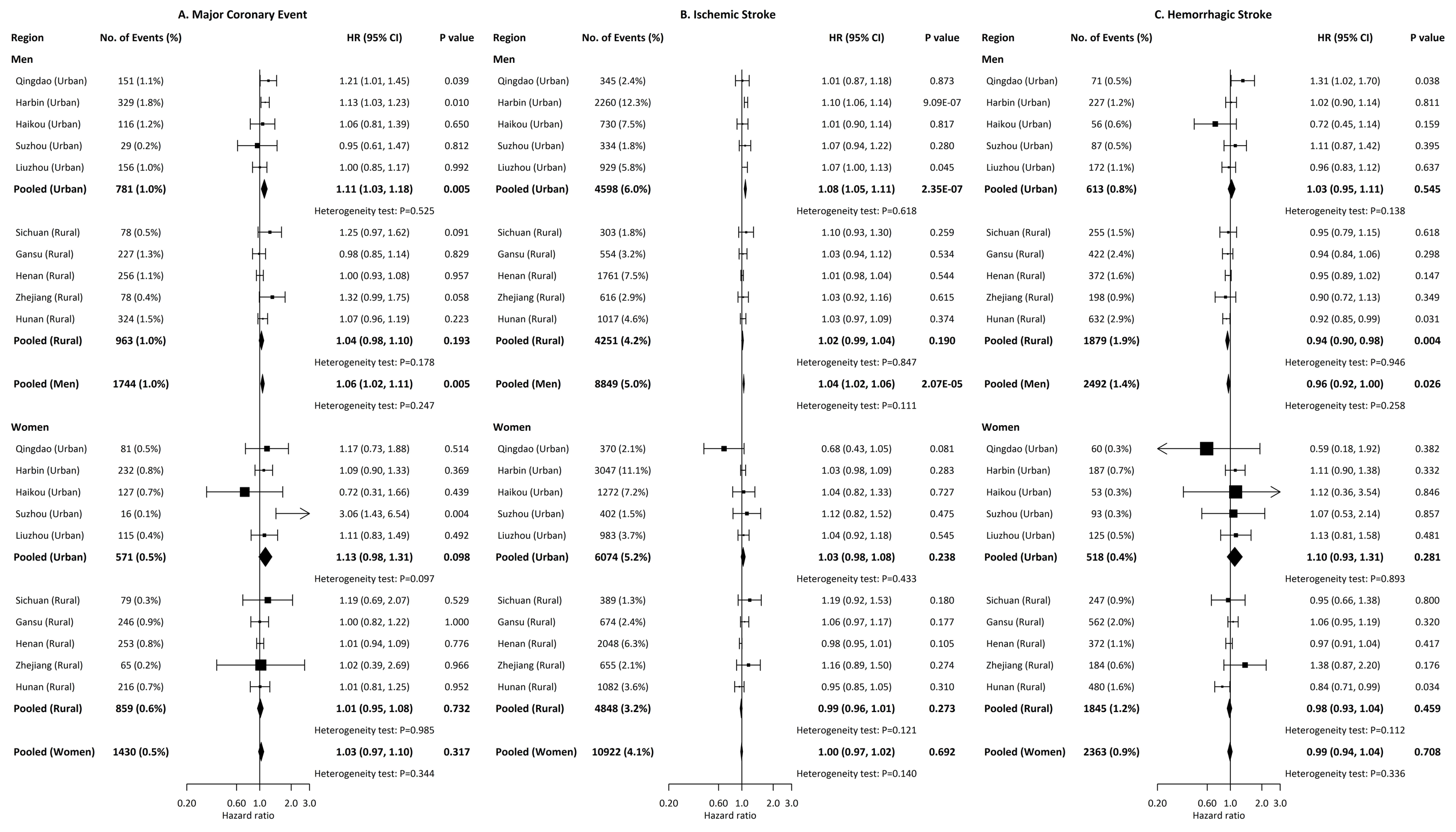


Supplementary Figure 8. The association of COex levels with future risk of major cardiovascular events across 10 study regions among men and women. The black boxes represent hazard ratios, with the size inversely proportional to the variance of the logarithm of the hazard ratio.

Hazard ratios were presented per 7 ppm (interquartile range) increase in COex levels; Cox models were stratified by age-at-risk (5-year group), and adjusted for BMI, systolic blood pressure, baseline status of diabetes (no/yes and treated/yes and untreated), baseline status of hypertension (no/yes and treated/yes and untreated), alcohol drinking, metabolic equivalent of a day’s work and leisure activities, levels of education and income, the survey season, whether smoked on the survey day, and smoking category, passive smoking, solid fuel use for cooking, solid fuel use for heating, slow burning of solid fuel and ventilation at home (yes/no). This graph was plotted with R 3.6.0 using the “forestplot” package (https://cran.r-project.org/web/packages/forestplot/index.html).





Supplementary Figure 9. The association of COex levels with future risk of major cardiovascular disease according to subgroups of baseline characteristics in never smokers.

Physical activity level was categorized according to Guidelines for data processing and analysis of the international physical activity questionnaire (IPAQ)-short and long forms (http://www.ipaq.ki.se/scoring.pdf). High physical activity was defined as vigorous-intensity activity achieving a minimum total physical activity of at least 1500 MET-minutes/week OR any combination of walking, moderate-intensity or vigorous-intensity activities achieving a minimum Total physical activity of at least 3000 MET-minutes/week; Moderate physical activity was defined as any combination of walking, moderate-intensity or vigorous intensity activities achieving a minimum total physical activity of at least 600 MET-minutes/week; Low physical activity was defined if not meeting criteria for "High" or "Moderate".

Cox models was stratified by age-at-risk (5-year group) and study areas (10 categories), and adjusted for BMI (continuous variable), systolic blood pressure (continuous variable), baseline status of diabetes (no/yes and treated/yes and untreated), baseline status of hypertension (no/yes and treated/yes and untreated), alcohol drinking (yes/no), metabolic equivalent of a day’s work and leisure activities (continuous variable), levels of education and income (each five categories), the survey season (four categories), passive smoking (yes/no), solid fuel use for cooking (yes/no), solid fuel use for heating (yes/no), slow burning of solid fuel (yes/no) and ventilation at home (yes/no). This graph was plotted with R 3.6.0 using the “forestplot” package (https://cran.r-project.org/web/packages/forestplot/index.html).

Supplementary Table S1. The association between COex levels and future risk of major cardiovascular disease among occasional smokers and ex regular smokers

| **Events** | | **COex quintiles** | | | | | | | | | | | | | | | | | | | |
| --- | --- | --- | --- | --- | --- | --- | --- | --- | --- | --- | --- | --- | --- | --- | --- | --- | --- | --- | --- | --- | --- |
|  |  | **Men** | | | | | | | | | |  | **Women** | | | | | | | | |
|  |  | **<2.0** | | | **2.0-3.0** | **3.0-5.0** | | **5.0-11.5** | | **≥11.5** | |  | **<2.0** | | **2.0-3.0** | | **3.0-5.0** | | **5.0-11.5** | | **≥11.5** |
| **Major Coronary Events** | | | | | | | | | | | | | | | | | | | | | |
| **Occasional smokers** | | | | | | | | | | | | | | | | | | | | | |
| No. of events (%) | | | 14 (0.6%) | | 19 (0.5%) | 29 (0.5%) | | 58 (0.9%) | | 11 (0.5%) | |  | 12 (1.2%) | | 6 (0.6%) | | 9 (0.7%) | 8 (0.8%) | | 4 (1.1%) | |
| Incidence rate | | | 35.9  (19.9, 64.5) | | 34.1  (20.4, 56.8) | 33.5  (21.6, 52.0) | | 62.2  (44.4, 87.2) | | 32.7  (17.7, 60.6) | |  | 41.4  (19.0, 90.5) | | 22.2  (8.3, 59.6) | | 31.2  (12.6, 77.2) | 34.3  (11.9, 99.5) | | 72.7  (24.3, 217.2) | |
| HR (95%CI) | | | Ref | | 0.92  (0.45, 1.86) | 0.73  (0.38, 1.43) | | 1.05  (0.55, 2.00) | | 0.44  (0.18, 1.04) | |  | Ref | | 0.17  (0.05, 0.58) | | 0.31  (0.10, 0.93) | 0.20  (0.06, 0.69) | | 0.35  (0.07, 1.85) | |
| **Ex regular smokers** | | | | | | | | | | | | | | | | | | | | | |
| No. of events (%) | | | 27 (0.9%) | | 56 (1.4%) | 73 (1.1%) | | 84 (1.5%) | | 14 (0.9%) | |  | 5 (1.2%) | 9 (2.3%) | | 7 (1.4%) | | 10 (3.1%) | | 0 | |
| Incidence rate | | | 69.3  (46.2, 103.9) | | 108.2  (79, 148.2) | 96.2  (73.3, 126.1) | | 131.2  (102.1, 168.5) | | 82.8  (48.1, 142.6) | |  | - | - | | - | | - | | - | |
| HR (95%CI) | | | Ref | | 1.29  (0.81, 2.08) | 0.97  (0.61, 1.56) | | 1.04  (0.64, 1.68) | | 0.53  (0.26, 1.10) | |  | Ref | 1.00  (0.30, 3.41) | | 0.65  (0.18, 2.33) | | 0.93  (0.27, 3.20) | | - | |
| **Ischemic Stroke** | | | | | | | | | | | | | | | | | | | | | |
| **Occasional smokers** | | | | | | | | | | | | | | | | | | | | | |
| No. of events (%) | 95 (3.9%) | | | 114 (3.1%) | | 272 (4.6%) | | 328 (5.1%) | | 142 (6.2%) | |  | 28 (2.8%) | 35 (3.5%) | | 48 (4 .0% ) | | 51 (4.8%) | | 23 (6.2%) | |
| Incidence rate | 363.4  (296.1, 446.0) | | | 294.0  (242.6, 356.4) | | 451.0  (396.1, 513.4) | | 511.3  (453.7, 576.2) | | 608.2  (510.2, 725.0) | |  | 281.5  (192.8, 410.9) | 366.2  (259.6, 516.6) | | 447.9  (330.2, 607.4) | | 588.7  (441.3, 785.2) | | 882.2  (587.1, 1326.0) | |
| HR (95%CI) | Ref | | | 0.78  (0.59, 1.02) | | 1.01  (0.79, 1.30) | | 0.98  (0.77, 1.27) | | 0.98  (0.73, 1.32) | |  | Ref | 0.90  (0.53, 1.52) | | 0.82  (0.49, 1.37) | | 0.76  (0.45, 1.30) | | 1.02  (0.53, 1.97) | |
| **Ex regular smokers** | | | | | | | | | | | | | | | | | | | | | |
| No. of events (%) | 175 (6.1%) | | | 259 (6.3%) | | 445 (7.0%) | | 434 (7.8%) | | 146 (9.4%) | |  | 18 (4.4%) | 37 (9.4%) | | 42 (8.6%) | | 47 (14.5%) | | 11 (16.9%) | |
| Incidence rate | 590.0  (507.4, 686.1) | | | 637.9  (560.7, 725.8) | | 742.8  (671.4, 821.7) | | 860.7  (777.3, 953.0) | | 1096.0  (928.1, 1294.0) | |  | 524.8  (330.8, 832.6) | 1131.0  (813.8, 1571.0) | | 1044.0  (754.7, 1444.0) | | 1697.0  (1256.0, 2292.0) | | 2420,0  (1372.0, 4267.0) | |
| HR (95%CI) | Ref | | | 0.92  (0.76, 1.12) | | 0.93  (0.77, 1.13) | | 0.86  (0.71, 1.05) | | 0.96  (0.74, 1.24) | |  | Ref | 1.39  (0.77, 2.52) | | 1.13  (0.62, 2.03) | | 1.15  (0.63, 2.09) | | 2.21  (0.96, 5.07) | |
| **Hemorrhagic Stroke** | | | | | | | | | | | | | | | | | | | | | |
| **Occasional smokers** | | | | | | | | | | | | | | | | | | | | | |
| No. of events (%) | 21 (0.9%) | | | 29 (0.8%) | | | 77 (1.3%) | | 93 (1.4%) | | 34 (1.5%) |  | 14 (1.4%) | 9 (0.9%) | | 10 (0.8%) | | 13 (1.2%) | | 3 (0.8%) | |
| Incidence rate | 81.4  (52.8, 125.6) | | | 76.2  (51.9, 112) | | | 129.6  (100.9, 166.6) | | 145.3  (115.7, 182.4) | | 146.5  (102.7, 209.0) |  | 111.7  (61.3, 203.7) | 75.0  (37.2, 151.3) | | 75.8  (37.1, 154.6) | | 123.0  (68.3, 221.7) | | 98.0  (31.1, 309.0) | |
| HR (95%CI) | Ref | | | 0.82  (0.46, 1.46) | | | 1.22  (0.74, 2.01) | | 1.00  (0.59, 1.67) | | 0.90  (0.49, 1.66) |  | Ref | 0.64  (0.27, 1.53) | | 0.88  (0.37, 2.10) | | 1.27  (0.51, 3.14) | | 0.77  (0.17, 3.52) | |
| **Ex regular smokers** | | | | | | | | | | | | | | | | | | | | | |
| No. of events (%) | 43 (1.5%) | | | | 74 (1.8%) | | 100 (1.6%) | | 100 (1.8%) | | 30 (1.9%) |  | 17 (4.1%) | 9 (2.3%) | | 12 (2.5%) | | 11 (3.4%) | | 2 (3.1%) | |
| Incidence rate | 157.2  (115.3, 214.4) | | | | 195.6  (154.9, 247.1) | | 177.9  (144.5, 219.1) | | 209.2  (169.6, 258.1) | | 233.6  (160.9, 339.1) |  | 491.2  (311.2, 775.2) | 265.0  (129.0, 544.6) | | 285.9  (151.3, 540.5) | | 374.2  (204.7, 684.1) | | 410.4  (102.1, 1650.0) | |
| HR (95%CI) | Ref | | | | 1.24  (0.84, 1.83) | | 1.07  (0.73, 1.57) | | 1.00  (0.67, 1.49) | | 0.85  (0.49, 1.45) |  | Ref | 0.62  (0.26, 1.49) | | 0.67  (0.29, 1.55) | | 0.92  (0.35, 2.43) | | 0.70  (0.06, 8.84) | |

Age-adjusted incidence rates were presented per 100,000 person-years;

Hazard ratios for developing cardiovascular events were calculated with Cox regression models stratified by age-at-risk (5-year group) and study areas (10 categories), and adjusted for BMI, systolic blood pressure, baseline status of diabetes, baseline status of hypertension, alcohol drinking, metabolic equivalent of a day’s work and leisure activities, levels of education and income, the survey season, passive smoking, solid fuel use for cooking, solid fuel use for heating, slow burning of solid fuel and ventilation at home, plus whether having smoked at least 100 cigarettes or equivalent and whether smoked on the survey day in occasional smokers, and plus cigarette equivalents per day, the time in years since smoking cessation and depth of inhalation in ex regular smokers.

The number of cigarette equivalents smoked per day was calculated for current and ex-regular smokers including all types of tobacco, with one pipe or one hand-rolled cigarette being treated as equal to 5/3 cigarettes, and one cigar being treated as equal to 2 cigarettes.

Supplementary Table S2. The association between COex levels and cardiovascular events after excluding events occurring within the first 2 years after baseline survey

| **Events** | **COex quintiles (ppm)** | | | | | | | | | | | |
| --- | --- | --- | --- | --- | --- | --- | --- | --- | --- | --- | --- | --- |
|  | **Men** | | | | |  | | **Women** | | | | |
|  | **<2.0** | **2.0-3.0** | **3.0-5.0** | **5.0-11.5** | **≥11.5** |  | | **<2.0** | **2.0-3.0** | **3.0-5.0** | **5.0-11.5** | **≥11.5** |
| **Major Coronary Events** | | | | | | | | | | | | |
| No. of events (%) | 68 (4.8%) | 116 (8.1%) | 217 (15.2%) | 442 (30.9%) | 586 (41.0%) |  | | 255 (21.5%) | 214 (18.1%) | 290 (24.5%) | 284 (24.0%) | 141 (11.9%) |
| Incidence rate | 38.4  (30.1, 49.1) | 22.0  (18.9, 25.6) | 315.6  (287.3, 346.6) | 262.6  (250.1, 275.7) | 76.7  (63.1, 93.1) |  | | 22.0  (18.9, 25.6) | 315.6  (287.3, 346.6) | 262.6  (250.1, 275.7) | 76.7  (63.1, 93.1) | 64.1  (58, 70.8) |
| Model 1 | Ref | 1.23  (0.91, 1.66) | 1.35  (1.03, 1.77) | 1.84  (1.42, 2.38) | 2.51  (1.94, 3.24) |  | | Ref | 0.92  (0.77, 1.11) | 1.18  (0.99, 1.40) | 1.43  (1.20, 1.71) | 1.57  (1.26, 1.95) |
| Model 2 | Ref | 1.09  (0.80, 1.47) | 1.03  (0.78, 1.37) | 1.26  (0.97, 1.65) | 1.60  (1.22, 2.10) |  | | Ref | 0.85  (0.70, 1.02) | 0.98  (0.81, 1.17) | 1.07  (0.89, 1.29) | 1.16  (0.91, 1.48) |
| Model 3 | Ref | 1.07  (0.79, 1.44) | 0.98  (0.74, 1.29) | 1.02  (0.78, 1.35) | 1.10  (0.82, 1.48) |  | | Ref | 0.84  (0.69, 1.01) | 0.96  (0.80, 1.15) | 0.98  (0.80, 1.18) | 0.96  (0.75, 1.24) |
| Model 4 | Ref | 1.06  (0.79, 1.44) | 0.97  (0.73, 1.29) | 1.02  (0.77, 1.34) | 1.09  (0.81, 1.47) |  | | Ref | 0.84  (0.69, 1.01) | 0.95  (0.79, 1.14) | 0.97  (0.80, 1.17) | 0.94  (0.73, 1.22) |
| **Ischemic Stroke** | | | | | | | | | | | | |
| No. of events (%) | 443 (6.0%) | 620 (8.4%) | 1274 (17.3%) | 2111 (28.7%) | 2912 (39.6%) |  | 1758 (18.8%) | | 1878 (20.0%) | 2325 (24.8%) | 2240 (23.9%) | 1166 (12.4%) |
| Incidence rate | 315.6  (287.3, 346.6) | 262.6  (250.1, 275.7) | 76.7  (63.1, 93.1) | 64.1  (58, 70.8) | 49.6  (40.8, 60.2) |  | 262.6  (250.1, 275.7) | | 76.7  (63.1, 93.1) | 64.1  (58.0, 70.8) | 49.6  (40.8, 60.2) | 54.9  (47.5, 63.3) |
| Model 1 | Ref | 0.96  (0.85, 1.08) | 1.13  (1.01, 1.26) | 1.29  (1.16, 1.43) | 1.76  (1.59, 1.95) |  | Ref | | 1.03  (0.96, 1.10) | 1.15  (1.08, 1.22) | 1.46  (1.37, 1.56) | 1.86  (1.72, 2.02) |
| Model 2 | Ref | 0.96  (0.85, 1.09) | 1.05  (0.94, 1.18) | 1.08  (0.97, 1.20) | 1.33  (1.19, 1.48) |  | Ref | | 1.08  (1.01, 1.16) | 1.05  (0.99, 1.13) | 1.05  (0.98, 1.13) | 1.11  (1.02, 1.22) |
| Model 3 | Ref | 0.95  (0.84, 1.08) | 1.02  (0.91, 1.14) | 0.99  (0.88, 1.10) | 1.13  (1.00, 1.28) |  | Ref | | 1.08  (1.01, 1.16) | 1.05  (0.98, 1.13) | 1.03  (0.96, 1.11) | 1.07  (0.98, 1.18) |
| Model 4 | Ref | 0.95  (0.84, 1.08) | 1.02  (0.91, 1.14) | 0.99  (0.88, 1.10) | 1.13  (1.00, 1.28) |  | Ref | | 1.08  (1.01, 1.16) | 1.05  (0.98, 1.12) | 1.03  (0.96, 1.11) | 1.06  (0.97, 1.17) |
| **Hemorrhagic Stroke** | | | | | | | | | | | | |
| No. of events (%) | 107 (5.5%) | 179 (9.2%) | 353 (18.1%) | 601 (30.7%) | 715 (36.6%) |  | | 422 (21.9%) | 440 (22.9%) | 448 (23.3%) | 393 (20.4%) | 222 (11.5%) |
| Incidence rate | 76.7  (63.1, 93.1) | 64.1  (58.0, 70.8) | 49.6  (40.8, 60.2) | 54.9  (47.5, 63.3) | 70.0  (62.9, 77.9) |  | | 64.1  (58.0, 70.8) | 49.6  (40.8, 60.2) | 54.9  (47.5, 63.3) | 70.0  (62.9, 77.9) | 91.9  (83.7, 100.9) |
| Model 1 | Ref | 1.29  (1.02, 1.64) | 1.48  (1.19, 1.85) | 1.46  (1.19, 1.80) | 1.59  (1.29, 1.96) |  | | Ref | 1.17  (1.02, 1.34) | 1.17  (1.02, 1.34) | 1.16  (1.01, 1.34) | 1.17  (0.98, 1.39) |
| Model 2 | Ref | 1.12  (0.88, 1.43) | 1.18  (0.95, 1.47) | 1.05  (0.85, 1.30) | 1.05  (0.85, 1.31) |  | | Ref | 1.07  (0.93, 1.22) | 1.00  (0.87, 1.15) | 0.95  (0.82, 1.11) | 1.12  (0.93, 1.36) |
| Model 3 | Ref | 1.11  (0.88, 1.42) | 1.16  (0.93, 1.45) | 0.99  (0.79, 1.23) | 0.94  (0.74, 1.20) |  | | Ref | 1.07  (0.93, 1.22) | 1.00  (0.87, 1.15) | 0.95  (0.81, 1.11) | 1.12  (0.92, 1.36) |
| Model 4 | Ref | 1.12  (0.88, 1.42) | 1.17  (0.93, 1.46) | 0.99  (0.79, 1.24) | 0.95  (0.75, 1.21) |  | | Ref | 1.07  (0.93, 1.22) | 1.00  (0.87, 1.15) | 0.95  (0.82, 1.11) | 1.13  (0.92, 1.38) |

Age-adjusted incidence rates were presented per 100,000 person-years;

Hazard ratios for fatal cardiovascular events were calculated with Cox regression models;

Model 1 was stratified by age-at-risk (5-year group), and urban residency, and adjusted for BMI (continuous variable), systolic blood pressure (continuous variable), baseline status of diabetes (no/yes and treated/yes and untreated), baseline status of hypertension (no/yes and treated/yes and untreated), alcohol drinking (yes/no), metabolic equivalent of a day’s work and leisure activities (continuous variable), levels of education and income (each five categories), and the survey season (four categories);

Model 2 was stratified by stratified by age-at-risk (5-year group) and study areas (10 categories) instead of urban residency, and adjusted for BMI (continuous variable), systolic blood pressure (continuous variable), baseline diabetes (yes/no), alcohol drinking (yes/no), metabolic equivalent of a day’s work and leisure activities (continuous variable), levels of education and income (each five categories), and the survey season (four categories);

Model 3 was further adjusted for smoking-related variables, including smoking status (four categories), whether having smoked on the survey day (yes/no), and passive smoking;

Model 4 was further adjusted for solid fuel use-related variables, including solid fuel use for cooking (yes/no), solid fuel use for heating (yes/no), slow burning of solid fuel (yes/no) and ventilation at home (yes/no)

Supplementary Table S3. The association of COex levels with fatal cardiovascular events

| **Events** | **COex quintiles** | | | | | | | | | | |
| --- | --- | --- | --- | --- | --- | --- | --- | --- | --- | --- | --- |
|  | **Men** | | | | |  | **Women** | | | | |
|  | **<2.0** | **2.0-3.0** | **3.0-5.0** | **5.0-11.5** | **≥11.5** |  | **<2.0** | **2.0-3.0** | **3.0-5.0** | **5.0-11.5** | **≥11.5** |
| **Total CCVD death** | | | | | | | | | | | |
| No. of events (%) | 194 (5.8%) | 272 (8.2%) | 534 (16.1%) | 1131 (34%) | 1191 (35.9%) |  | 639 (22.2%) | 643 (22.3%) | 662 (23%) | 599 (20.8%) | 336 (11.7%) |
| Incidence rate | 30.5  (23.8, 39.0) | 15.9  (13.5, 18.7) | 41.0  (32.4, 51.8) | 33.1  (29.0, 37.8) | 88.3  (76.0, 102.6) |  | 15.9  (13.5, 18.7) | 41.0  (32.4, 51.8) | 33.1  (29.0, 37.8) | 88.3  (76.0, 102.6) | 56.4  (51.3, 62.0) |
| Basic model | Ref | 0.90  (0.75, 1.09) | 0.94  (0.8, 1.12) | 1.07  (0.91, 1.25) | 1.07  (0.91, 1.26) |  | Ref | 1.03  (0.92, 1.15) | 0.98  (0.88, 1.10) | 0.96  (0.85, 1.09) | 1.05  (0.89, 1.23) |
| Full model | Ref | 0.90  (0.75, 1.08) | 0.93  (0.78, 1.10) | 0.99  (0.84, 1.16) | 0.93  (0.78, 1.11) |  | Ref | 1.03  (0.92, 1.15) | 0.97  (0.87, 1.09) | 0.93  (0.82, 1.05) | 0.97  (0.82, 1.14) |
| **Fatal Ischaemic Events** | | | | | | | | | | | |
| No. of events (%) | 18 (5.2%) | 26 (7.5%) | 46 (13.3%) | 131 (37.8%) | 126 (36.3%) |  | 58 (21.4%) | 68 (25.1%) | 57 (21%) | 54 (19.9%) | 34 (12.5%) |
| Incidence rate | 41.0  (32.4, 51.8) | 33.1  (29.0, 37.8) | 88.3  (76.0, 102.6) | 56.4  (51.3, 62.0) | 34.5  (28, 42.5) |  | 33.1  (29.0, 37.8) | 88.3  (76.0, 102.6) | 56.4  (51.3, 62.0) | 34.5  (28.0, 42.5) | 48.6  (42, 56.3) |
| Basic model | Ref | 0.85  (0.63, 1.16) | 1.00  (0.76, 1.31) | 1.15  (0.89, 1.49) | 1.18  (0.91, 1.54) |  | Ref | 1.05  (0.87, 1.26) | 1.01  (0.84, 1.21) | 1.02  (0.84, 1.24) | 1.20  (0.95, 1.52) |
| Full model | Ref | 0.85  (0.62, 1.15) | 0.97  (0.74, 1.27) | 1.03  (0.79, 1.34) | 0.96  (0.73, 1.28) |  | Ref | 1.04  (0.87, 1.25) | 1.00  (0.83, 1.20) | 0.97  (0.80, 1.18) | 1.09  (0.85, 1.39) |
| Fatal Haemorrhagic Stroke | | | | | | | | | | | |
| No. of events (%) | 79 (6.6%) | 102 (8.5%) | 183 (15.2%) | 419 (34.7%) | 423 (35.1%) |  | 287 (27.1%) | 244 (23%) | 230 (21.7%) | 200 (18.9%) | 99 (9.3%) |
| Incidence rate | 88.3  (76.0, 102.6) | 56.4  (51.3, 62.0) | 34.5  (28.0, 42.5) | 48.6  (42.0, 56.3) | 65.0  (58.3, 72.5) |  | 56.4  (51.3, 62.0) | 34.5  (28.0, 42.5) | 48.6  (42.0, 56.3) | 65.0  (58.3, 72.5) | 74.1  (67.0, 82.1) |
| Basic model | Ref | 0.86  (0.64, 1.16) | 0.84  (0.64, 1.10) | 0.90  (0.70, 1.17) | 0.84  (0.65, 1.09) |  | Ref | 0.91  (0.76, 1.08) | 0.86  (0.72, 1.03) | 0.88  (0.72, 1.06) | 0.97  (0.74, 1.27) |
| Full model | Ref | 0.87  (0.65, 1.17) | 0.85  (0.65, 1.11) | 0.89  (0.68, 1.16) | 0.81  (0.61, 1.08) |  | Ref | 0.91  (0.77, 1.08) | 0.86  (0.72, 1.03) | 0.86  (0.71, 1.05) | 0.95  (0.72, 1.25) |

Age-adjusted incidence rates were presented per 100 000 person-years;

Hazard ratios for fatal CCVD events were calculated with Cox regression models;

Basic model was stratified by age-at-risk (5-year group), and urban residency (rural/urban), and adjusted for BMI, systolic blood pressure, baseline status of diabetes (no/yes and treated/yes and untreated), baseline status of hypertension (no/yes and treated/yes and untreated), alcohol drinking, metabolic equivalent of a day’s work and leisure activities, levels of education and income, and the survey season;

Full model was stratified by age-at-risk (5-year group) and study areas (10 categories), and further adjusted for passive smoking, solid fuel use for cooking, solid fuel use for heating, slow burning of solid fuel and ventilation at home, plus whether smoked on the survey day, and smoking status (four categories) in addition to the basic model.

*P*_trend_ of the association of COex with total CCVD death and fatal ischemic event in basic model among men were 0.049 and 0.015, respectively.
